# Clozapine prescribing in Germany: temporal trends and regional variations, 2012–2022

**DOI:** 10.1038/s41537-026-00763-w

**Published:** 2026-05-14

**Authors:** Oliver H. F. Scholle, Oliver Riedel, Mishal Qubad, Michael Dörks, Bianca Kollhorst, Robert A. Bittner, Christian J. Bachmann

**Affiliations:** 1 Department of Clinical Epidemiology, Leibniz Institute for Prevention Research and Epidemiology – BIPS, Bremen, Germany; 2 Goethe University Frankfurt, University Hospital, Department of Psychiatry, Psychosomatic Medicine and Psychotherapy, Frankfurt, Germany; 3 Ernst Strüngmann Institute of the Max Planck Society, Frankfurt, Germany; 4 Department of Health Services Research, Carl von Ossietzky Universität Oldenburg, Oldenburg, Germany; 5 Department of Statistical Methods in Epidemiology, Leibniz Institute for Prevention Research and Epidemiology – BIPS, Bremen, Germany; 6 Department of Child and Adolescent Psychiatry, Ulm University, Ulm, Germany

**Keywords:** Schizophrenia, Psychosis

## Abstract

Clozapine is the most effective and only approved drug for treatment-resistant schizophrenia (TRS). Studies based on data up to 2014 concluded that it is underutilized in most industrialized countries, including Germany. Since 2019, national guidelines have explicitly been recommending clozapine as first-line therapy in TRS. We aimed to assess whether clozapine use in Germany has increased in recent years and to examine regional variations. Using claims data covering about 20% of the German population (GePaRD), we calculated the yearly prescription prevalence and incidence of clozapine among individuals aged 0–64 years based on outpatient dispensations. For 2022, we also assessed regional variations in clozapine prescription prevalence at the district level (restricted to *N* = 202 districts with ≥20,000 individuals). From 2012 to 2022, the overall (age- and sex-standardized) prescription prevalence of clozapine continuously decreased by 16% (from 77.6 to 65.5 per 100,000). The relative decline was greatest in women aged 30–39 years (-51%) and in men aged 30–34 years (-57%), in urban areas (large urban cities: -23%; urban districts: -16%), and in regions with high socioeconomic status (-22%). Over the same period, the overall prescription incidence of clozapine decreased by 41%. In 2022, regional clozapine prescription prevalence differed up to 39-fold. In conclusion, clozapine prescribing in Germany did not increase from 2012 to 2022, despite new clozapine-favoring guidelines, and showed substantial regional variation. Our results suggest a persisting underutilization of clozapine in most of Germany. Further research on barriers and facilitators for clozapine use in Germany is needed.

## Introduction

Clozapine remains the most effective antipsychotic for schizophrenia, and the only medication specifically approved for patients with treatment-resistant schizophrenia (TRS)^1^. At least one-third of all individuals with schizophrenia fulfill TRS criteria^2–4^. These patients bear a disproportionately high share of the disorder’s stigma, clinical burden, and associated healthcare costs^5^, highlighting the need for adequate evidence-based treatment with clozapine.

Both meta-analyses of randomized controlled trials and real-world cohort studies consistently demonstrate clozapine’s unique clinical benefits^6–14^. Among all antipsychotics, clozapine shows the largest improvements in global psychopathology and in positive, negative, and depressive symptoms^15–18^. Compared to other antipsychotics, clozapine elicits greater improvements in treatment adherence and lower rates of suicidal behavior^7,8,19–22^. Clozapine is also the most effective drug for reducing aggressive behavior, substance use, hospitalization rates, and for preventing further relapses in patients with a second psychotic episode^23–28^.

Moreover, clozapine achieves greater reductions in all-cause and suicide mortality than any other antipsychotic in patients with schizophrenia^10,11,18^. Clozapine’s singular effect on all-cause mortality is partly attributable to its superior impact on treatment adherence for somatic comorbidities^29^. Further, clozapine has a lower risk of extrapyramidal side-effects^7^. Accordingly, current expert consensus guidelines recommend clozapine as a second-line treatment in cases of persistent positive symptoms accompanied by suicidality, aggression, extrapyramidal symptoms, or tardive dyskinesia^30^.

Crucially, a substantial proportion of TRS cases manifest during the first episode of psychosis (FEP), with approximately 25% of FEP patients meeting TRS criteria^3^. Delayed initiation of clozapine after the emergence of treatment-resistance—typically averaging 5–7 years—substantially lowers response rates^31–33^. This underscores the critical importance of a timely detection of treatment resistance and immediate initiation of clozapine, already during the early stages of manifest illness.

However, contrary to national and international guideline recommendations^34–36^, clozapine remains considerably underutilized across most healthcare systems. Estimates from international studies suggest that in most countries, only a minority of eligible patients receive clozapine, with considerable variations both within and across countries^1,37^.

Among the available metrics to quantify drug utilization, prescription prevalence (i.e., the number of persons with at least one prescription in a population) is essential. The most recent population-based study on trends in the prescription prevalence of clozapine, encompassing 17 countries including Germany, covered the period from 2005 to 2014^37^. Since then, long-term trend data on the prescription prevalence of clozapine have not been systematically investigated across Europe. Moreover, only few studies have examined fine-grained regional variation in clozapine prescribing.

Therefore, our aim was to comprehensively characterize longitudinal trends in outpatient clozapine prescribing in Germany from 2012 to 2022—both overall and stratified by age, sex, urbanicity, and area-level socioeconomic deprivation—and to quantify the extent of regional variations at the district level.

## Methods

We conducted year-wise cross-sectional studies, analyzing routinely collected German healthcare data.

### Data source

We used claims data from four statutory health insurance providers in Germany (GePaRD), which include information on approximately 25 million persons who have been insured with one of the participating providers since 2004 or later^38^. Per calendar year, the data cover approximately 20% of the general population, representing all geographical regions of Germany. Available demographic data include sex, age, and district-level region of residence. Prescription data encompass all reimbursed medications prescribed in the outpatient setting by general practitioners or specialists. Prescriptions are coded according to the German modification of the WHO Anatomical Therapeutic Chemical (ATC) classification system (version as of April 2023 for this study).

### Study design

To be eligible for the year-wise study populations from 2012 to 2022, individuals had to meet the following criteria: (a) valid information on sex and an age between 0 and 64 years, (b) documented residency in Germany and (c) continuous insurance coverage throughout the respective calendar year, allowing for coverage gaps of up to 30 days. Individuals who died or were born during the respective year were also included, provided they were continuously insured from January 1 until their date of death or from birth until December 31. For estimating the prescription incidence, eligible individuals additionally had to be continuously insured during the entire preceding calendar year and must not have received a clozapine prescription during that year. Persons aged 65 years or older were excluded to minimize the likelihood of capturing clozapine prescriptions for conditions such as psychosis related to Parkinson’s disease or various forms of dementia, which are more prevalent in older age groups^37^.

### Clozapine prescriptions

For each year, we identified prescriptions of clozapine (ATC code N05AH02) based on reimbursed outpatient dispensations.

### Urbanicity and socioeconomic deprivation

District-level characteristics were linked to individuals via their region of residence (total number of districts: 401). Urbanicity was defined according to the official classification of district types based on settlement structure (as of 2017), categorized into four levels: “large urban city” (≥100,000 inhabitants), “urban district” (≥50% of the population in large and medium-sized cities and population density ≥150 inhabitants/km², or corresponding density excluding these cities), “rural district with densification tendencies” (≥50% population in large and medium-sized cities but density <150 inhabitants/km², or <50% with density excluding these cities ≥100 inhabitants/km²), and “sparsely populated rural district” (<50% population in large and medium-sized cities and density excluding these cities <100 inhabitants/km²)^39^. Additionally, we analyzed trends by district-level socioeconomic deprivation using the publicly available German Index of Socioeconomic Deprivation (as of 2018)^40^.

### Data analysis

For each year from 2012 to 2022, we calculated the prescription prevalence of clozapine as the number of individuals with at least one prescription per 100,000 individuals. Using the same approach, we also calculated the prescription incidence, restricting the analysis to individuals with continuous insurance coverage (as previously defined) and no clozapine prescription in the preceding calendar year. All estimates were computed both overall and stratified by sex and age group. For prescription prevalence, additional stratifications were performed by urbanicity and district-level socioeconomic deprivation, the latter classified into quintiles; with the second to fourth quintile combined into a single intermediate category.

For the year 2022, we calculated the clozapine prescription prevalence at the district level. Due to small sample sizes, we limited the analysis to districts with a database population of at least 20,000 individuals, resulting in a total of 202 (out of 401) districts.

All prevalence and incidence estimates were calculated along with 95% confidence intervals (CIs) and were directly standardized by age and sex, using the German population of December 31, 2022 as the reference. All statistical analyses were performed using SAS version 9.4 (SAS Institute, Cary, NC, USA).

## Results

From 2012 to 2022, the number of included individuals per year ranged from 11,653,312 (2012) to 13,671,786 (2022) (Supplementary Table S1).

### Temporal trends overall and by age and sex

**Fig. 1:**
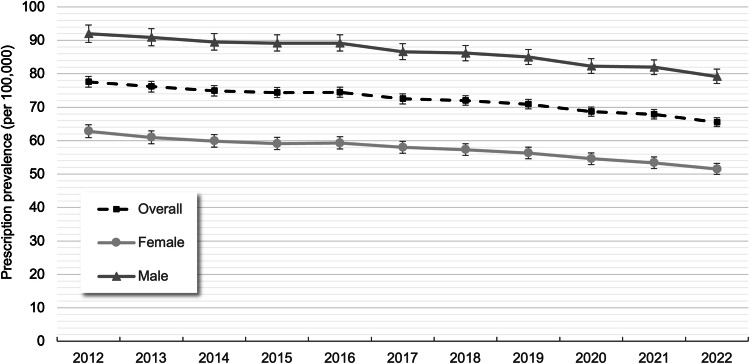
Standardized prescription prevalence (with 95% CIs) of clozapine overall and by sex between 2012 and 2022.

In 2022, the overall age- and sex-standardized clozapine prescription prevalence was 65.5 per 100,000 (95% CI: 64.2; 66.9) (Fig. 1 and Supplementary Table S1). Prevalence was higher among males (79.2 per 100,000; 95% CI: 77.0; 81.3) than in females (51.5 per 100,000; 95% CI: 49.8; 53.2).

**Fig. 2:**
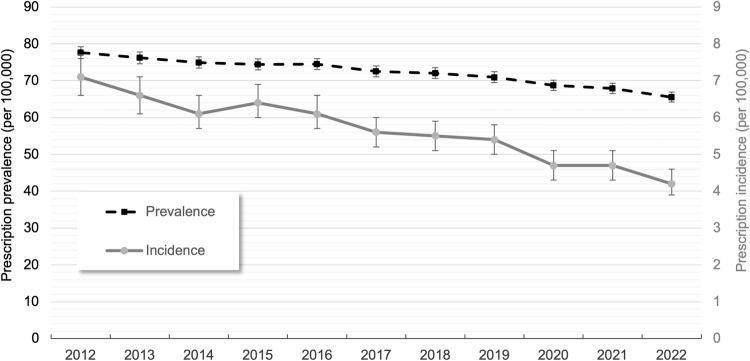
Overall age- and sex-standardized prescription prevalence and incidence (with 95% CIs) of clozapine between 2012 and 2022.

Between 2012 and 2022, the overall age- and sex-standardized prescription prevalence of clozapine continuously decreased (−16%, from 77.6 to 65.5 per 100,000) (Fig. 1). This downward trend was observed in both sexes, with an 18% decrease among females (from 62.8 to 51.5 per 100,000) and a 14% decrease among males (from 92.0 to 79.2 per 100,000). As shown in Fig. 2, the age- and sex-standardized prescription incidence declined even more sharply than the prevalence. Between 2012 and 2022, the prescription incidence of clozapine decreased by 41%, from 7.1 to 4.2 per 100,000 (Supplementary Table S2).

**Fig. 3:**
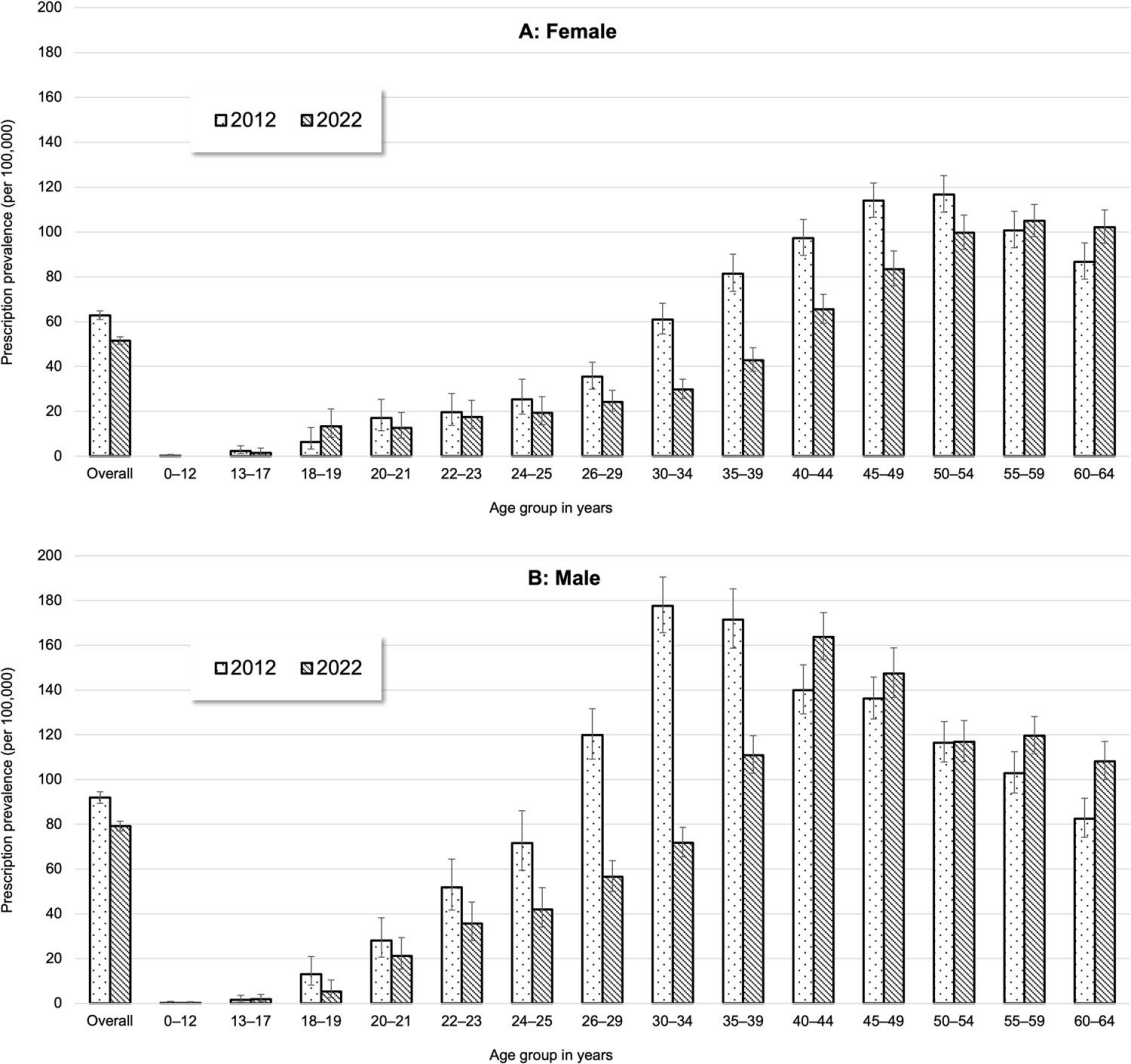
Prescription prevalence (with 95% CIs) of clozapine by age group among females (**A)** and males (**B)** in 2012 and 2022.

Among females, in 2022, the prescription prevalence of clozapine increased with age, peaking at approximately 100 per 100,000 in the age groups from 50 to 64 years (Fig. 3A and Supplementary Table S1). Between 2012 and 2022, the most pronounced relative declines in prevalence among women occurred in the age groups 30–34 (−51%) and 35–39 (−47%) years. Among males, in 2022, the prescription prevalence of clozapine also increased with age, peaking at approximately 160 per 100,000 in the age group 40–44 years (before declining in older age groups; Fig. 3B and Supplementary Table S1). Between 2012 and 2022, the most pronounced relative declines in prevalence among men occurred in the age groups 26–29 (−48%) and 30–34 (−57%) years.

### Temporal trends by urbanicity and socioeconomic deprivation, and district-level variation

**Fig. 4:**
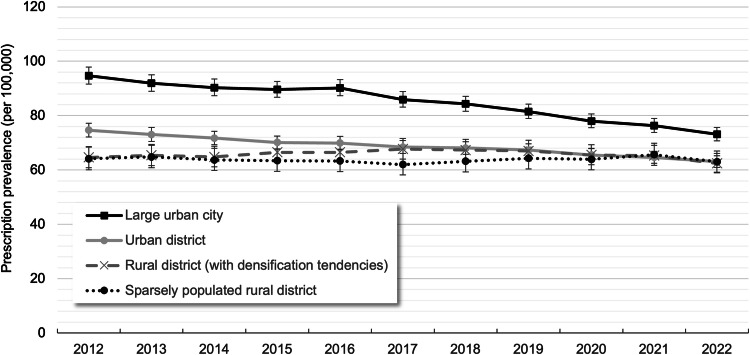
Age- and sex-standardized prescription prevalence (with 95% CIs) of clozapine by urbanicity between 2012 and 2022.

In 2022, the age- and sex-standardized prescription prevalence of clozapine was highest in large urban cities at 73.1 per 100,000 (95% CI: 70.7; 75.7), compared to urban districts (63.0 per 100,000 [95% CI: 60.9; 65.2]), rural districts (62.4 per 100,000 [95% CI: 58.9; 66.1]), and sparsely populated rural districts (63.0 per 100,000 [95% CI: 59.2; 67.1]) (Fig. 4 and Supplementary Table S3). Between 2012 and 2022, the prescription prevalence declined in large urban cities (−23%) and urban districts (−16%) and remained largely stable in rural districts.

Between 2012 and 2022, the age- and sex-standardized clozapine prescription prevalence declined across all strata of district-level socioeconomic deprivation, with the steepest decline observed in the least deprived areas. In the quintile representing the least deprived districts, the prevalence decreased by 22% (from 88.7 to 69.2 per 100,000). In the middle three quintiles (second to fourth), the decline was 14% (from 74.7 to 64.2 per 100,000), and in the most deprived quintile, it was 9% (from 72.6 to 66.3 per 100,000) (Supplementary Fig. S1 and Table S3).

Of the 401 German districts, we included only those with a database population of ≥20,000 individuals in 2022 (*n* = 202; Table 1 and Supplementary Table S4). These districts account for approximately 73% of the total German population^41^. As indicated by the 5th and 95th percentile, the 5% of the districts with the lowest prescription prevalence of clozapine had values ≤ 29.7 per 100,000, while the top 5% had values ≥ 139.3 per 100,000, corresponding to a 4.7-fold difference. The full range spanned from 5.4 to 209 per 100,000, corresponding to a 38.7-fold difference in clozapine prescription prevalence between districts.

**Table 1:** Distribution of the standardized prescription prevalence of clozapine across 202 districts in 2022 (per 100,000 individuals).

Distribution statistics	Prevalence per 100,000 (*n* = 202 districts)
Median	57.9
First quartile; third quartile	42.3; 77.5
5th percentile; 95th percentile	29.7; 139.3
Minimum; maximum	5.4; 209.0

Estimates of the prevalence proportion of clozapine prescriptions are age- and sex-standardized to the population of Germany as of December 31, 2022.

The sample included 202 (out of 401) districts with ≥20,000 individuals in the database population. Based on the total population of these districts in official statistics, they represent approximately 73% of the overall German population in 2022.

## Discussion

From 2012 to 2022, we found a decline in outpatient clozapine prescribing in Germany. This decline was mainly due to (a) a decrease in the number of individuals newly initiated on clozapine, (b) less prescribing among women aged 30–39 years and men aged 26–34 years, and (c) reduced prescribing in urban areas and in regions with higher socioeconomic status. In addition, we observed substantial regional variation in clozapine prescribing at the district level.

Our study is the first to update trends on the prescription prevalence of clozapine in a European country since the international analysis of 17 countries from 2005 to 2014^37^. In that study, clozapine prescription prevalence in Germany increased between 2005 and 2010, then plateaued at approximately 95 per 100,000 individuals until 2014. The authors concluded that clozapine remained underutilized in many countries and emphasized the need for targeted interventions to promote its use^37^. Extending these findings, our study shows that clozapine use in Germany did not increase beyond 2014; instead, it declined steadily until 2022, with no indication of a new plateau.

Based on the same data source as the present study, a recent publication showed a continuous decrease in the prevalence of treated schizophrenia between 2012 and 2021 (−9%)^42^. Data on temporal trends in the prevalence of TRS are lacking. However, assuming that the proportion of TRS among patients with schizophrenia remains stable, a decline in schizophrenia prevalence would be expected to translate into a declining prevalence of TRS. Hence, the decrease in clozapine prescribing seen in the present study might at least partly be attributable to a decrease in the prevalence of TRS in our population. Changes in the organization of mental health care may also have influenced clozapine prescribing over time. However, data on such changes during the study period are unavailable, precluding conclusions on this point. Data on the prescription prevalence of antipsychotics in general in Germany for the same period as in this study are also unavailable. However, based on defined daily doses, overall antipsychotic prescribing in Germany slightly increased between 2014 and 2023^43^. This indicates that the decrease in clozapine prescribing observed in our study is not necessarily attributable to a general trend of declining antipsychotic prescribing.

The estimated clozapine prescription prevalences in both studies presenting data for Germany (Bachmann et al. and ours) likely indicate clozapine underutilization, given that they are far below the expected prevalence estimates for TRS. With 12-month prevalence estimates of schizophrenia ranging from 330 to 460 per 100,000 in the general population^44,45^ and 36.7% of all individuals with schizophrenia fulfilling TRS criteria^2^, the corresponding 12-month prevalence of TRS, i.e., the expected prescription prevalence of clozapine, can be estimated at approximately 121 to 169 per 100,000.

The lower clozapine prescription prevalence observed during the overlapping years 2012 to 2014 in our study (<80 per 100,000), compared to approximately 95 per 100,000 reported by Bachmann et al. ^37^, may be attributed to two key differences: first, our study excluded individuals aged ≥65 years; second, there were differences in the socioeconomic characteristics—and therefore the underlying schizophrenia risk—of the study populations covered by the respective statutory health insurance providers^46^. Despite this discrepancy in overall prevalence, the sex-specific age patterns observed in our study closely mirror those reported by Bachmann et al. ^37^ for Germany.

The more pronounced decline in clozapine prescribing incidence compared to the prevalence observed in our study indicates marked changes in prescribing patterns over the study period. First, substantially fewer individuals were newly started on clozapine, defined as clozapine initiation following a clozapine-free period of at least one year. Second, among those already receiving clozapine, the average treatment duration appears to have increased. This interpretation is supported by the established relationship between prevalence, incidence, and duration^47^. If prescription incidence declines more sharply than prescription prevalence, this implies that treated individuals remain on treatment (here: clozapine) for longer, thereby slowing the decline in prevalence relative to incidence. The reasons for the decrease in clozapine initiation remain unclear, especially as the key position of clozapine in the treatment of TRS has continually been strengthened through clinical guidelines and quality assurance measures. Qualitative studies drawing on prescribers’ attitudes towards clozapine in TRS might help to elucidate the motives behind this lamentable trend.

To our knowledge, no study so far has examined the association between clozapine prescribing and urbanicity or socioeconomic deprivation in the general population. Among studies focusing specifically on patients with schizophrenia, clozapine use has been reported to be higher in urban areas compared to rural areas^48^, while another study found no association with socioeconomic deprivation^49^. However, these findings are not directly comparable to results based on general population data, as the risk of schizophrenia itself varies by both urbanicity^50^ and socioeconomic status^51,52^. Notably, in contrast to the general risk of schizophrenia, higher urbanicity has been associated with a lower risk of TRS among patients with schizophrenia^53,54^. Given that the design of these studies already accounts for regional differences in the risk of schizophrenia, these findings do not imply a higher clozapine prescribing prevalence in rural areas compared to urban areas in the general population. Regardless of the complex association of schizophrenia and TRS with urbanicity, our findings demonstrate that the observed overall decline in clozapine prescription prevalence is primarily seen in urban regions and those with higher socioeconomic status.

Several recent studies have examined regional variations in clozapine prescribing among general, i.e. unrestricted, populations in countries such as the United States (US)^55,56^, the United Kingdom (UK)^57^, Japan^58^, and Norway^59^. While these studies reported substantial intra-country regional differences in prescribing patterns, these analyses were based on relatively large administrative units, such as 50 states in the US, 14 regions in England, or 19 counties in Norway. Our study examined prescribing patterns at a much finer spatial resolution, thus revealing even more pronounced regional variations—up to a 38-fold difference. Compared with findings from other countries, lower regional differences have been reported: up to 13-fold in the US in 2019^55^, 2.4-fold in the UK^57^, and 1.6-fold in Norway^59^. Variation of this magnitude is unlikely to be attributable solely to differences in the prevalence or burden of schizophrenia. Rather, it indicates significant inconsistencies in prescribing practices, which may reflect deviations from evidence-based care in the majority of studied regions.

Our observation of regional differences in clozapine prescribing, including higher prescribing in large urban cities, may, at least in part, be explained by differences in the availability and distribution of residential care facilities for patients with severe mental illness. These settings may concentrate patients with a more severe course of illness, including treatment-resistant schizophrenia, in large urban areas.

### Strengths and limitations

A key strength of our study lies in the use of a large sample of routinely collected healthcare data covering a substantial proportion of the general population in Germany. This enabled comprehensive, population-based analyses, while minimizing recall and non-responder bias. Moreover, we did not restrict our study to patients with schizophrenia, even though clozapine underutilization in this patient population was the focus of our study. Nevertheless, by restricting our study population to people below 65 years of age, most patients who might have been prescribed clozapine for other indications (e.g., psychosis in Parkinson’s disease), were likely excluded.

Our study also has several limitations. First, we could not capture clozapine prescriptions issued during inpatient stays. However, in the vast majority of cases, clozapine initiated during hospitalization is continued after discharge and thus captured in outpatient data, provided that the hospital stay does not exceed 12 months, which is extremely uncommon in Germany. Therefore, a substantial underestimation of clozapine use is unlikely. Second, although this study was based on a large data source, we did not include the whole German population. Third, in German claims data, patients’ prescriptions are not directly linked to corresponding diagnoses. Clozapine might therefore also have been prescribed to a relevant degree for indications other than TRS. However, this would even strengthen our conclusion that clozapine is underutilized. Finally, no information was available on the duration of untreated psychosis. If this duration decreased over the study period, the number of persons with TRS—and therefore those eligible for clozapine treatment—would also have decreased, which could at least partly explain the observed decline in clozapine prescribing.

### Implications

Assessing population-based trends and regional variation in clozapine prescribing using healthcare data is essential for evaluating its use in routine clinical practice. Such real-world data has the potential to reveal the extent of underutilization and help determine whether prescribing practices align with clinical needs. Analyses stratified by demographic characteristics may elucidate the drivers of temporal trends, while identifying regions with low prescribing rates can uncover potential access barriers. These data can inform policymakers and guide the development of targeted interventions for adequate clozapine utilization.

In our study, the steepest decrease in clozapine prescribing was observed among younger women and men. One possible reason for the decrease in younger people might be concerns regarding weight gain, a typical adverse effect of clozapine. This might have been reinforced by the increasing use of social media in the last decade, which has been linked to higher body image disturbance^60^. However, this interpretation is speculative, and our data do not allow conclusions regarding such an association. In the largest study examining patient satisfaction among clozapine users, weight gain—despite being the most frequently reported adverse drug reaction—was not associated with lower patient satisfaction^61^.

Our findings underscore the need for effective measures to promote evidence-based prescribing of clozapine for people with TRS in Germany—a need that likely extends to many other countries as well. Barriers to more widespread use of clozapine appear to be predominantly prescriber-related^1,62–64^. This includes factors such as disproportionate concerns about safety and complexity of monitoring, as well as systemic limitations and stigmatization of schizophrenia among mental health professionals^1,5^. Moreover, psychiatrists frequently anticipate low patient acceptance of clozapine^63^. A recent survey of board-certified psychiatrists with extensive experience in clozapine use practicing in inpatient and outpatient care in psychiatric hospitals confirmed these findings for Germany^65^. Increasing reluctance of prescribers to even offer clozapine could contribute to the decline in clozapine initiation in younger patients, evident in our data. This is not only clinically unjustified but ethically problematic^66^ as patients might often not even be adequately informed about clozapine as a vital treatment option^67,68^ despite clear evidence of high patient satisfaction, treatment preference, and adherence once it is initiated^61,69,70^.

Emphasizing the patients’ perspective and needs through shared decision-making constitutes a critical yet underused tool that significantly improves the likelihood of clozapine recommendations and addresses misalignments between prescriber beliefs and patient preferences^1,71,72^. To this end, patient advocacy should also be crucial^73^—especially for reducing the considerable stigma associated with TRS^5^—but remains under-investigated.

Perhaps most importantly, clozapine’s superior efficacy for reducing mortality in TRS should be sufficient to motivate its use^1^. Mortality reduction is an unequivocal treatment priority in other medical disciplines. Adopting this strategy for psychiatry should help to facilitate adequate clozapine utilization. To this end, introducing formalized and mandatory training regimes for psychiatrists regarding all aspects of clozapine use will be essential^74^. This should be complemented by the systematic and widespread establishment of specialized TRS treatment teams^1^.

Ideally linked to psychiatric hospitals with extensive expertise in clozapine use, TRS teams integrated with community services have been shown to substantially increase timely outpatient access to clozapine^75,76^. Beyond facilitating clozapine initiation, TRS teams can serve as hubs for disseminating expertise in the detection and management of TRS and for formally training prescribers^1^.

While our results suggest that treatment initiation might face the greatest barriers, successful clozapine initiation and its continued use also require effective prevention and management of adverse effects^6^. Accordingly, side-effect management should be a central focus of formalized training in clozapine use. By offering practical training in optimizing clozapine efficacy and managing adverse effects, as well as providing consultation during and after treatment initiation, TRS teams may reduce prescriber-related barriers and thereby facilitate broader implementation of clozapine in both inpatient and outpatient settings^1,75,76^.

Other systemic barriers also need to be addressed. Strict blood monitoring requirements constitute an important barrier both from the patient and prescriber perspectives^1^. One important and recently realized measure has been a regulatory label change for clozapine by the European Medicines Agency (EMA), following longstanding demands from experts, including the European Clozapine Task Force^77^. The previous requirement of lifelong monthly blood monitoring was not evidence-based and increasingly viewed as outdated and burdensome^78^. As of 2025, EMA recommends reducing the frequency of blood monitoring to once every 12 weeks after the first treatment year without neutropenia, and to annual checks after two years, focusing solely on the absolute neutrophil count (ANC) rather than total leukocyte counts. This revision is expected to improve access to clozapine for individuals with TRS, reduce stigma and logistical barriers, and potentially save lives^77,78^.

A recent global Delphi consensus aligns with these recommendations, advocating for lower ANC thresholds for clozapine cessation and discontinuation of routine absolute neutrophil count monitoring after 2 years^72^. The panel further emphasizes the need for quarterly comprehensive adverse drug reaction monitoring and simplified protocols to reduce patient burden. These evidence-based measures offer a more practical and patient-centered approach to clozapine safety, aiming to enhance outcomes for patients with TRS. Moreover, the reduced costs resulting from these changes should free resources urgently needed to improve other crucial aspects of care for patients with TRS^79^.

## Conclusions

Clozapine prescribing in Germany did not increase from 2012 to 2022, despite new clozapine-favoring guidelines, and showed substantial regional variation. Our results suggest a persisting underutilization of clozapine in most of Germany. Further research on barriers and facilitators for clozapine use in Germany is needed.

### Preprint disclosure

A preprint version of this manuscript has been posted on medRxiv (URL: https://www.medrxiv.org/content/10.64898/2025.12.03.25341492v1). The copyright holder for this preprint is the author, who has granted medRxiv a license to display the preprint in perpetuity. All rights reserved. No reuse allowed without permission.

### Ethical standards

In Germany, the utilization of health insurance data for scientific research is regulated by the Code of Social Law. All involved health insurance providers, as well as the Federal Office for Social Security and the Senator for Health, Women and Consumer Protection in Bremen, as their responsible authorities, approved the use of data for this study. Informed consent for studies based on claims data is required by law unless obtaining consent appears unacceptable and would bias results, which was the case in this study. According to the Ethics Committee of the University of Bremen, studies based solely on pseudonymized personal data are exempt from institutional review board review.

## Supplementary information

Supplementary Material

## Data Availability

As we are not the owners of the data, we are not legally entitled to grant access to the data. In accordance with German data protection regulations, access to the data is granted only to employees of the Leibniz Institute for Prevention Research and Epidemiology – BIPS on the BIPS premises and in the context of approved research projects. Third parties may only access the data in cooperation with BIPS and after signing an agreement for guest researchers at BIPS.
