# Supplementary material for: Clozapine prescribing in Germany: temporal trends and regional variations, 2012–2022
Source: Schizophrenia (Heidelb). 2026 May 14;12(1):44. doi: 10.1038/s41537-026-00763-w (PMC13176340; doi:10.1038/s41537-026-00763-w)
Supplement: Supplementary file 1 — Supplementary Material [file 41537_2026_763_MOESM1_ESM.pdf]

SUPPLEMENTARY MATERIAL

Clozapine prescribing in Germany: temporal trends and regional variations, 2012–2022

Oliver H. F. Scholle, Oliver Riedel, Mishal Qubad, Michael Dörks, Bianca Kollhorst, Robert A. Bittner[†], and Christian J. Bachmann[†]

[†] These authors contributed equally and share last authorship.

Corresponding author:

Christian J. Bachmann

E-mail: christian.bachmann@uniklinik-ulm.de

TABLE OF CONTENTS

Figure S1: Age- and sex-standardized prescription prevalence (with 95% CIs) of clozapine by district-level socioeconomic deprivation between 2012 and 2022 2

Table S1: Standardized prescription prevalence of clozapine by age and sex for each calendar year from 2012 to 2022 (per 100,000 persons; 95% confidence intervals in brackets) 3

Table S2: Standardized prescription incidence of clozapine by age and sex for each calendar year from 2012 to 2022 (per 100,000 persons; 95% confidence intervals in brackets) 7

Table S3: Standardized prescription prevalence of clozapine (age 0–64 years) by regional characteristics for each calendar year from 2012 to 2022 (per 100,000 persons; 95% confidence intervals in brackets) 9

Table S4: Prescription prevalence of clozapine (age 0–64 years) by district among the 202 districts with a database population of ≥20,000 persons in 2022 (per 100,000 persons), in ascending order of the standardized prevalence....................10

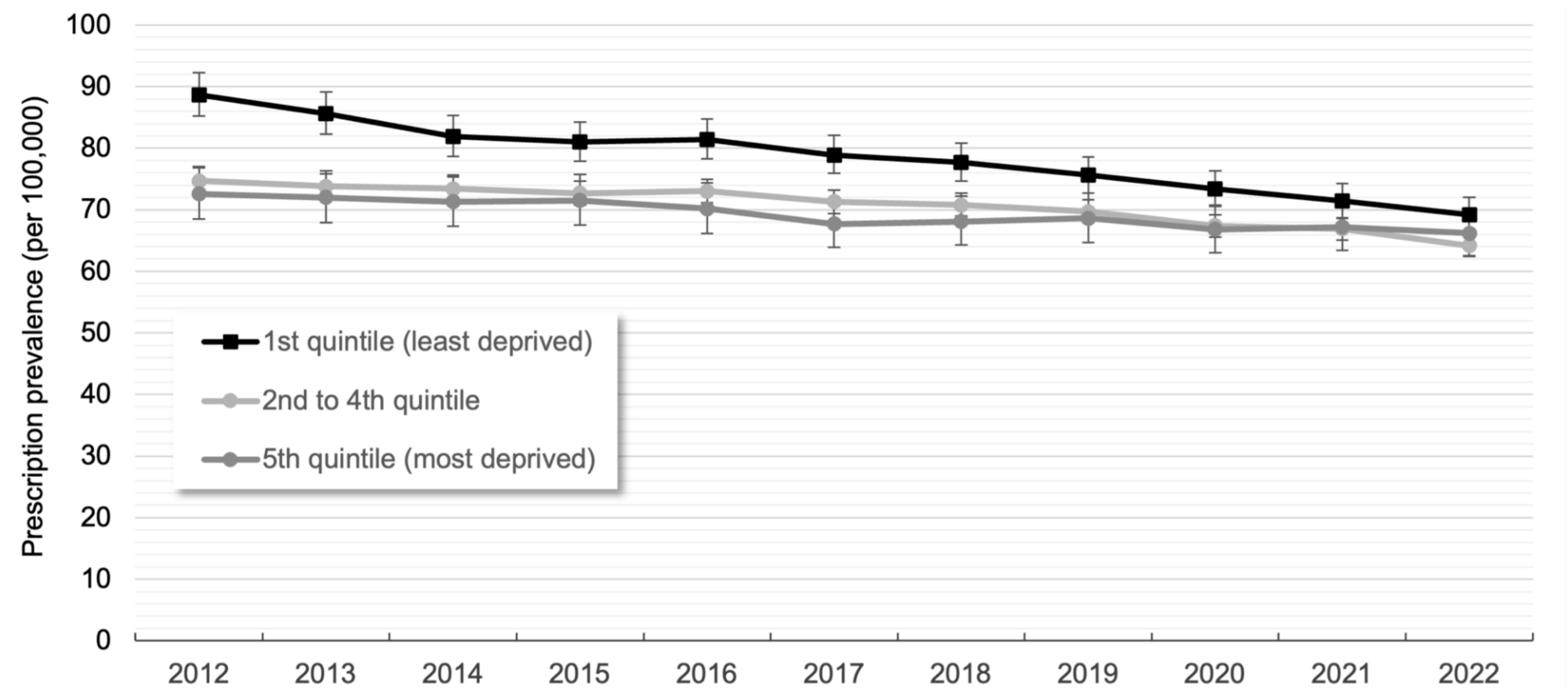

Figure S1: Age- and sex-standardized prescription prevalence (with 95% CIs) of clozapine by district-level socioeconomic deprivation between 2012 and 2022

Table S1: Standardized prescription prevalence of clozapine by age and sex for each calendar year from 2012 to 2022 (per 100,000 persons; 95% confidence intervals in brackets)

	**2012**	**2013**	**2014**	**2015**	**2016**
**Total number of (database) population, 0–64 years, n**	11,653,312	12,133,011	12,374,789	12,661,181	12,735,204
Total number of (database) population, all age years, n	14,677,493	15,241,994	15,570,274	15,948,015	16,079,136
Overall, all age years	81.4 (80.0; 83.0)	79.9 (78.4; 81.4)	78.4 (77.0; 79.8)	77.3 (75.9; 78.7)	77.6 (76.2; 79.0)
**Overall, 0–64 years**	77.6 (76.0; 79.2)	76.2 (74.6; 77.8)	74.9 (73.4; 76.5)	74.4 (72.9; 75.9)	74.5 (73.0; 76.0)
Female, 0–64 years	62.8 (60.9; 64.8)	61.0 (59.1; 62.9)	59.9 (58.1; 61.8)	59.1 (57.3; 61.0)	59.3 (57.5; 61.2)
Male, 0–64 years	92.0 (89.4; 94.6)	90.9 (88.4; 93.5)	89.5 (87.1; 92.0)	89.2 (86.8; 91.6)	89.2 (86.8; 91.7)
**Both sexes aged 0–17 years**	0.6 (0.4; 1.0)	0.7 (0.5; 1.1)	0.8 (0.5; 1.2)	0.7 (0.5; 1.1)	0.5 (0.3; 0.8)
Females aged 0–17 years	0.7 (0.4; 1.3)	1.0 (0.6; 1.7)	1.0 (0.6; 1.7)	0.6 (0.3; 1.2)	0.5 (0.2; 1.1)
Males aged 0–17 years	0.5 (0.2; 1.1)	0.5 (0.2; 1.0)	0.6 (0.3; 1.2)	0.8 (0.5; 1.4)	0.5 (0.2; 1.0)
**Both sexes aged 18–64 years**	98.7 (96.7; 100.8)	96.9 (94.9; 98.9)	95.3 (93.3; 97.3)	94.6 (92.6; 96.5)	94.8 (92.9; 96.7)
Females aged 18–64 years	79.6 (77.1; 82.1)	77.2 (74.9; 79.7)	75.8 (73.5; 78.2)	74.9 (72.6; 77.3)	75.2 (72.9; 77.5)
Males aged 18–64 years	117.5 (114.2; 120.8)	116.1 (112.9; 119.4)	114.3 (111.2; 117.5)	113.8 (110.7; 116.9)	113.9 (110.9; 117.0)
**Both sexes by age group (years)**					
0–12	0.1 (0.0; 0.4)	0.2 (0.1; 0.6)	0.1 (0.0; 0.5)	0.1 (0.0; 0.4)	0
13–17	1.9 (1.1; 3.2)	2.2 (1.4; 3.5)	2.6 (1.6; 4.0)	2.4 (1.5; 3.8)	1.8 (1.1; 3.1)
18–19	9.8 (6.6; 14.5)	11.7 (8.4; 16.3)	10.2 (7.2; 14.5)	10.5 (7.5; 14.6)	9.8 (7.0; 13.8)
20–21	22.7 (17.8; 29.1)	19.5 (15.2; 25.0)	17.2 (13.2; 22.4)	23.0 (18.4; 28.9)	18.3 (14.3; 23.4)
22–23	36.5 (30.3; 43.9)	30.1 (24.8; 36.6)	33.2 (27.5; 40.0)	30.1 (24.8; 36.5)	32.7 (27.2; 39.3)
24–25	49.5 (42.3; 58.0)	50.7 (43.8; 58.6)	42.8 (36.6; 50.1)	40.1 (34.2; 47.1)	39.8 (33.8; 46.9)
26–29	79.3 (73.0; 86.1)	66.0 (60.4; 72.1)	62.0 (56.8; 67.8)	54.2 (49.5; 59.5)	50.0 (45.5; 54.9)
30–34	120.8 (113.8; 128.2)	113.5 (106.9; 120.5)	104.9 (98.7; 111.5)	96.6 (90.8; 102.9)	89.4 (83.9; 95.3)
35–39	127.0 (119.4; 135.1)	126.1 (118.7; 133.9)	125.4 (118.3; 133.0)	125.4 (118.5; 132.7)	123.7 (117.0; 130.8)
40–44	118.6 (112.0; 125.6)	116.7 (110.0; 123.9)	117.1 (110.2; 124.4)	117.4 (110.5; 124.8)	122.0 (114.9; 129.6)
45–49	125.0 (119.1; 131.3)	124.6 (118.7; 130.9)	122.3 (116.4; 128.6)	119.0 (113.1; 125.3)	117.3 (111.2; 123.7)
50–54	116.6 (110.7; 122.9)	116.6 (110.8; 122.7)	115.9 (110.3; 121.9)	117.6 (112.0; 123.5)	120.1 (114.5; 126.0)
55–59	101.8 (95.8; 108.1)	105.0 (99.1; 111.3)	102.1 (96.4; 108.2)	103.5 (97.8; 109.4)	107.8 (102.1; 113.8)
60–64	84.6 (78.8; 90.7)	85.4 (79.8; 91.5)	90.6 (84.8; 96.8)	96.3 (90.4; 102.6)	99.6 (93.6; 105.9)

	2012	2013	2014	2015	2016
**Female by age group (years)**					
0–12	0.1 (0.0; 0.8)	0.4 (0.1; 1.1)	0.2 (0.1; 1.0)	0.1 (0.0; 0.8)	0
13–17	2.3 (1.1; 4.5)	2.6 (1.4; 4.9)	2.9 (1.6; 5.2)	1.9 (0.9; 3.9)	1.9 (0.9; 4.0)
18–19	6.4 (3.2; 12.8)	8.8 (5.1; 15.2)	6.6 (3.5; 12.2)	9.9 (6.1; 16.1)	12.3 (8.0; 19.1)
20–21	17.0 (11.4; 25.4)	15.4 (10.3; 23.0)	11.9 (7.6; 18.7)	11.0 (6.9; 17.4)	9.5 (5.8; 15.5)
22–23	19.6 (13.8; 27.9)	20.1 (14.4; 28.0)	21.5 (15.5; 29.8)	18.0 (12.6; 25.6)	16.1 (11.1; 23.3)
24–25	25.4 (18.7; 34.3)	24.1 (18.0; 32.3)	22.8 (16.9; 30.7)	26.2 (19.9; 34.6)	25.7 (19.3; 34.2)
26–29	35.5 (29.9; 42.0)	28.3 (23.5; 34.0)	28.2 (23.5; 33.8)	26.3 (21.9; 31.5)	26.4 (22.0; 31.6)
30–34	61.0 (54.4; 68.3)	57.7 (51.5; 64.7)	53.2 (47.3; 59.8)	43.5 (38.3; 49.4)	43.8 (38.6; 49.7)
35–39	81.4 (73.5; 90.1)	73.4 (66.1; 81.5)	74.5 (67.3; 82.4)	74.4 (67.4; 82.1)	67.8 (61.2; 75.0)
40–44	97.2 (89.5; 105.7)	93.1 (85.4; 101.6)	89.0 (81.3; 97.5)	88.0 (80.3; 96.5)	90.6 (82.6; 99.3)
45–49	113.9 (106.5; 121.8)	111.3 (104.1; 119.2)	107.8 (100.6; 115.6)	103.0 (95.8; 110.7)	100.6 (93.3; 108.5)
50–54	116.7 (108.9; 125.2)	116.2 (108.6; 124.4)	112.9 (105.5; 120.8)	111.6 (104.4; 119.2)	111.8 (104.6; 119.4)
55–59	100.7 (92.9; 109.2)	104.2 (96.4; 112.7)	102.9 (95.3; 111.1)	104.7 (97.3; 112.8)	105.8 (98.4; 113.8)
60–64	86.6 (78.9; 95.0)	83.9 (76.6; 92.0)	87.9 (80.4; 96.1)	93.3 (85.6; 101.6)	99.6 (91.7; 108.2)
**Male by age group (years)**					
0–12	0.1 (0.0; 0.8)	0	0	0	0
13–17	1.5 (0.6; 3.6)	1.8 (0.8; 3.7)	2.3 (1.2; 4.4)	3.0 (1.7; 5.2)	1.7 (0.8; 3.7)
18–19	13.0 (8.1; 20.9)	14.4 (9.5; 21.8)	13.7 (9.0; 20.8)	11.0 (7.0; 17.3)	7.4 (4.3; 12.8)
20–21	28.1 (20.6; 38.3)	23.3 (16.9; 32.1)	22.1 (16.0; 30.7)	34.4 (26.6; 44.5)	26.6 (20.0; 35.4)
22–23	51.9 (41.8; 64.6)	39.3 (31.0; 49.9)	43.9 (35.0; 55.2)	41.2 (32.7; 51.9)	47.9 (38.7; 59.3)
24–25	71.6 (59.5; 86.1)	74.9 (63.3; 88.6)	61.1 (50.9; 73.4)	52.8 (43.4; 64.2)	52.7 (43.2; 64.3)
26–29	119.9 (109.1; 131.7)	100.9 (91.2; 111.6)	93.4 (84.4; 103.4)	80.2 (72.1; 89.2)	71.9 (64.4; 80.3)
30–34	177.6 (165.6; 190.4)	166.4 (155.1; 178.5)	154.0 (143.5; 165.4)	147.1 (137.0; 158.0)	132.6 (123.2; 142.9)
35–39	171.4 (158.6; 185.1)	177.2 (164.6; 190.7)	174.8 (162.8; 187.8)	175.0 (163.3; 187.4)	178.1 (166.6; 190.3)
40–44	139.9 (129.3; 151.3)	140.2 (129.3; 151.9)	145.0 (133.8; 157.1)	146.6 (135.3; 158.9)	153.3 (141.7; 165.7)
45–49	136.2 (127.1; 145.9)	137.9 (128.7; 147.8)	136.8 (127.6; 146.7)	135.0 (125.7; 145.1)	133.9 (124.4; 144.2)
50–54	116.5 (107.8; 125.9)	117.0 (108.5; 126.2)	119.0 (110.5; 128.0)	123.6 (115.2; 132.7)	128.4 (119.9; 137.6)
55–59	102.8 (94.0; 112.5)	105.8 (97.0; 115.3)	101.4 (93.0; 110.6)	102.2 (93.9; 111.1)	109.8 (101.3; 118.9)
60–64	82.5 (74.2; 91.7)	87.0 (78.6; 96.3)	93.3 (84.7; 102.8)	99.5 (90.7; 109.2)	99.5 (90.7; 109.2)

Estimates of the prevalence proportion of clozapine prescriptions are age- and sex-standardized to the population of Germany as of 31 December 2022.

Table S1 (continued): Standardized prescription prevalence of clozapine by age and sex for each calendar year from 2012 to 2022 (per 100,000 persons; 95% confidence intervals in brackets)

	2017	2018	2019	2020	2021	2022
**Total number of (database) population, 0–64 years, n**	12,988,508	13,155,467	13,291,386	13,477,263	13,507,154	13,671,786
Total number of (database) population, all age years, n	16,412,811	16,649,554	16,853,824	17,115,837	17,219,027	17,469,599
Overall, all age years	75.9 (74.6; 77.3)	75.0 (73.7; 76.4)	74.1 (72.8; 75.4)	72.5 (71.2; 73.8)	71.7 (70.4; 73.0)	69.4 (68.2; 70.6)
**Overall, 0–64 years**	72.5 (71.1; 74.0)	72.0 (70.5; 73.5)	70.9 (69.4; 72.3)	68.7 (67.3; 70.1)	67.9 (66.5; 69.3)	65.5 (64.2; 66.9)
Female, 0–64 years	58.0 (56.2; 59.8)	57.3 (55.6; 59.1)	56.3 (54.5; 58.0)	54.6 (52.9; 56.4)	53.4 (51.7; 55.1)	51.5 (49.8; 53.2)
Male, 0–64 years	86.6 (84.3; 89.0)	86.2 (84.0; 88.6)	85.0 (82.8; 87.3)	82.3 (80.1; 84.5)	82.0 (79.8; 84.2)	79.2 (77.0; 81.3)
**Both sexes aged 0–17 years**	0.5 (0.3; 0.8)	0.5 (0.3; 0.9)	0.5 (0.3; 0.9)	0.4 (0.2; 0.7)	0.5 (0.3; 0.8)	0.5 (0.3; 0.9)
Females aged 0–17 years	0.6 (0.3; 1.2)	0.6 (0.3; 1.2)	0.7 (0.4; 1.3)	0.5 (0.2; 1.1)	0.6 (0.3; 1.3)	0.4 (0.2; 1.0)
Males aged 0–17 years	0.4 (0.1; 0.9)	0.4 (0.2; 1.0)	0.4 (0.2; 0.9)	0.3 (0.1; 0.8)	0.3 (0.1; 0.8)	0.6 (0.3; 1.2)
**Both sexes aged 18–64 years**	92.3 (90.4; 94.2)	91.6 (89.8; 93.5)	90.2 (88.3; 92.0)	87.4 (85.6; 89.2)	86.4 (84.7; 88.2)	83.4 (81.6; 85.1)
Females aged 18–64 years	73.5 (71.3; 75.8)	72.6 (70.4; 74.9)	71.3 (69.1; 73.5)	69.2 (67.1; 71.5)	67.7 (65.6; 69.9)	65.3 (63.2; 67.4)
Males aged 18–64 years	110.7 (107.7; 113.7)	110.1 (107.2; 113.1)	108.6 (105.7; 111.6)	105.1 (102.3; 108.0)	104.8 (102.0; 107.6)	101.0 (98.3; 103.8)
**Both sexes by age group (years)**						
0–12	0	0	0	0.1 (0.0; 0.4)	0.1 (0.0; 0.4)	0.1 (0.0; 0.4)
13–17	1.7 (1.0; 3.0)	1.9 (1.1; 3.2)	1.9 (1.1; 3.3)	1.3 (0.7; 2.4)	1.6 (0.9; 2.8)	1.7 (1.0; 3.0)
18–19	11.2 (8.1; 15.4)	9.1 (6.4; 13.1)	8.5 (5.9; 12.5)	10.2 (7.1; 14.5)	9.3 (6.5; 13.5)	9.2 (6.3; 13.4)
20–21	19.6 (15.6; 24.8)	18.0 (14.2; 22.9)	20.1 (16.0; 25.4)	18.3 (14.3; 23.3)	19.4 (15.2; 24.7)	17.0 (13.1; 22.1)
22–23	30.4 (25.2; 36.8)	28.1 (23.2; 34.1)	29.9 (24.8; 35.9)	27.5 (22.7; 33.2)	26.1 (21.4; 31.8)	27.0 (22.2; 32.8)
24–25	35.1 (29.6; 41.7)	40.4 (34.4; 47.4)	38.3 (32.6; 45.1)	32.8 (27.6; 39.0)	32.2 (27.1; 38.2)	31.2 (26.2; 37.1)
26–29	46.6 (42.3; 51.4)	44.6 (40.4; 49.3)	44.3 (40.1; 48.9)	44.8 (40.5; 49.5)	44.7 (40.4; 49.4)	41.0 (37.0; 45.4)
30–34	80.2 (75.0; 85.7)	71.5 (66.8; 76.6)	65.5 (61.0; 70.3)	58.8 (54.7; 63.3)	54.3 (50.4; 58.6)	51.4 (47.5; 55.5)
35–39	116.3 (109.9; 122.9)	111.5 (105.5; 117.9)	103.2 (97.5; 109.3)	94.4 (89.0; 100.1)	88.5 (83.3; 94.0)	77.4 (72.6; 82.4)
40–44	124.5 (117.5; 132.0)	122.5 (115.6; 129.7)	124.2 (117.4; 131.3)	121.0 (114.5; 127.8)	120.4 (114.0; 127.0)	114.7 (108.7; 121.1)
45–49	113.8 (107.6; 120.3)	115.6 (109.1; 122.3)	114.8 (108.3; 121.7)	115.3 (108.7; 122.3)	116.3 (109.6; 123.4)	115.4 (108.8; 122.4)
50–54	118.7 (113.1; 124.5)	121.4 (115.7; 127.3)	118.8 (113.1; 124.8)	113.8 (108.1; 119.8)	110.2 (104.5; 116.2)	108.2 (102.4; 114.4)
55–59	108.8 (103.3; 114.7)	110.4 (104.9; 116.2)	112.1 (106.7; 117.8)	111.9 (106.5; 117.5)	115.1 (109.7; 120.8)	112.3 (107.0; 117.9)
60–64	98.4 (92.5; 104.6)	101.8 (95.9; 108.0)	102.2 (96.5; 108.3)	102.7 (97.1; 108.7)	104.3 (98.7; 110.2)	105.1 (99.6; 110.9)

	2017	2018	2019	2020	2021	2022
**Female by age group (years)**						
0–12	0	0	0	0.1 (0.0; 0.8)	0.1 (0.0; 0.8)	0
13–17	2.2 (1.1; 4.4)	2.2 (1.1; 4.4)	2.5 (1.3; 4.9)	1.4 (0.6; 3.5)	2.0 (1.0; 4.3)	1.5 (0.6; 3.5)
18–19	8.8 (5.2; 14.9)	7.1 (3.9; 12.8)	10.5 (6.5; 17.2)	11.0 (6.7; 18.0)	11.1 (6.8; 18.1)	13.4 (8.5; 21.0)
20–21	13.1 (8.7; 19.8)	13.1 (8.7; 19.7)	14.0 (9.4; 20.9)	14.9 (10.1; 22.1)	14.0 (9.3; 21.1)	12.6 (8.1; 19.5)
22–23	13.7 (9.2; 20.5)	15.0 (10.3; 21.9)	21.0 (15.3; 28.7)	18.7 (13.4; 26.1)	15.1 (10.4; 22.0)	17.5 (12.3; 24.9)
24–25	21.2 (15.5; 29.0)	20.4 (14.9; 28.1)	18.2 (13.0; 25.5)	18.0 (13.0; 25.1)	22.4 (16.7; 30.1)	19.3 (14.1; 26.6)
26–29	25.5 (21.3; 30.6)	25.1 (20.8; 30.2)	24.8 (20.6; 29.9)	25.3 (21.0; 30.5)	24.5 (20.3; 29.6)	24.2 (20.0; 29.3)
30–34	40.8 (35.8; 46.4)	38.7 (34.0; 44.1)	35.4 (31.0; 40.6)	33.5 (29.2; 38.3)	30.5 (26.5; 35.2)	29.8 (25.8; 34.4)
35–39	64.9 (58.6; 71.9)	62.8 (56.7; 69.6)	54.9 (49.3; 61.2)	46.3 (41.2; 52.1)	47.4 (42.2; 53.2)	42.8 (38.0; 48.3)
40–44	83.7 (76.1; 92.1)	78.9 (71.7; 87.0)	78.2 (71.1; 86.1)	78.3 (71.3; 86.0)	71.7 (65.1; 78.9)	65.5 (59.3; 72.3)
45–49	100.2 (92.7; 108.4)	98.3 (90.6; 106.7)	95.8 (88.0; 104.3)	93.8 (85.9; 102.4)	93.5 (85.5; 102.1)	83.4 (75.9; 91.5)
50–54	112.2 (105.0; 119.8)	111.0 (103.9; 118.7)	107.9 (100.8; 115.6)	101.1 (94.1; 108.7)	97.6 (90.5; 105.3)	99.6 (92.2; 107.5)
55–59	106.5 (99.2; 114.4)	108.2 (101.0; 116.0)	110.1 (102.9; 117.7)	107.8 (100.8; 115.3)	108.3 (101.3; 115.8)	104.9 (98.1; 112.3)
60–64	98.5 (90.8; 106.9)	100.5 (92.8; 108.8)	100.6 (93.0; 108.8)	103.8 (96.3; 111.9)	101.8 (94.5; 109.6)	102.1 (94.9; 109.8)
**Male by age group (years)**						
0–12	0	0	0	0	0	0.1 (0.0; 0.7)
13–17	1.3 (0.5; 3.1)	1.6 (0.7; 3.5)	1.4 (0.6; 3.3)	1.1 (0.4; 3.0)	1.1 (0.4; 2.9)	1.9 (0.9; 4.1)
18–19	13.4 (8.9; 20.2)	11.1 (7.1; 17.4)	6.7 (3.7; 12.0)	9.4 (5.7; 15.6)	7.7 (4.4; 13.5)	5.2 (2.6; 10.4)
20–21	25.7 (19.4; 34.1)	22.7 (16.8; 30.6)	25.9 (19.5; 34.3)	21.4 (15.6; 29.3)	24.4 (18.1; 32.9)	21.2 (15.3; 29.4)
22–23	45.7 (36.9; 56.7)	40.1 (32.0; 50.2)	38.0 (30.3; 47.6)	35.5 (28.1; 44.8)	36.1 (28.6; 45.7)	35.6 (28.1; 45.2)
24–25	47.8 (38.9; 58.8)	58.6 (48.7; 70.5)	56.7 (47.1; 68.4)	46.2 (37.8; 56.6)	41.1 (33.3; 50.8)	42.0 (34.1; 51.7)
26–29	66.2 (59.1; 74.2)	62.7 (55.8; 70.6)	62.3 (55.4; 70.1)	62.8 (55.8; 70.6)	63.4 (56.4; 71.3)	56.5 (50.0; 63.9)
30–34	117.5 (108.8; 127.0)	102.7 (94.7; 111.4)	94.0 (86.5; 102.1)	82.9 (76.0; 90.4)	76.9 (70.3; 84.1)	71.8 (65.5; 78.8)
35–39	166.1 (155.4; 177.5)	158.9 (148.7; 169.8)	150.1 (140.4; 160.5)	141.0 (131.7; 150.9)	128.4 (119.6; 137.8)	110.9 (102.8; 119.5)
40–44	165.1 (153.4; 177.7)	165.7 (154.3; 178.0)	169.8 (158.5; 181.9)	163.4 (152.7; 174.8)	168.7 (158.1; 180.1)	163.7 (153.5; 174.6)
45–49	127.3 (117.8; 137.6)	132.8 (122.7; 143.7)	133.7 (123.5; 144.9)	136.7 (126.3; 148.1)	139.1 (128.6; 150.5)	147.4 (136.8; 158.9)
50–54	125.2 (116.8; 134.2)	131.7 (123.0; 140.9)	129.7 (121.0; 139.0)	126.5 (117.7; 135.8)	122.6 (113.8; 132.1)	116.8 (108.0; 126.3)
55–59	111.1 (102.9; 120.1)	112.5 (104.4; 121.3)	114.1 (106.1; 122.8)	115.9 (107.9; 124.6)	122.0 (113.8; 130.7)	119.6 (111.6; 128.2)
60–64	98.3 (89.6; 107.7)	103.2 (94.5; 112.7)	103.9 (95.4; 113.3)	101.7 (93.4; 110.7)	106.9 (98.6; 115.9)	108.2 (100.0; 117.0)

Estimates of the prevalence proportion of clozapine prescriptions are age- and sex-standardized to the population of Germany as of 31 December 2022.

Table S2: Standardized prescription incidence of clozapine by age and sex for each calendar year from 2012 to 2022 (per 100,000 persons; 95% confidence intervals in brackets)

	2012	2013	2014	2015	2016
**Total number of (database) population, 0–64 years, n**	10,161,228	11,308,968	11,754,464	11,974,203	12,071,904
**Overall, 0–64 years**	7.1 (6.6; 7.7)	6.6 (6.1; 7.1)	6.1 (5.6; 6.5)	6.4 (5.9; 6.9)	6.1 (5.7; 6.6)
Female, 0–64 years	6.0 (5.4; 6.7)	5.4 (4.8; 6.0)	4.7 (4.2; 5.3)	4.9 (4.4; 5.5)	4.9 (4.4; 5.5)
Male, 0–64 years	8.2 (7.4; 9.1)	7.8 (7.0; 8.6)	7.4 (6.7; 8.2)	7.8 (7.1; 8.5)	7.3 (6.6; 8.0)
**Both sexes aged 0–17 years**	0.4 (0.2; 0.7)	0.4 (0.2; 0.8)	0.4 (0.2; 0.8)	0.4 (0.3; 0.8)	0.2 (0.1; 0.4)
Females aged 0–17 years	0.4 (0.2; 1.1)	0.4 (0.2; 1.0)	0.5 (0.2; 1.1)	0.3 (0.1; 0.8)	0.2 (0.1; 0.7)
Males aged 0–17 years	0.3 (0.1; 0.9)	0.4 (0.2; 1.0)	0.4 (0.2; 0.9)	0.6 (0.3; 1.1)	0.1 (0.0; 0.6)
**Both sexes aged 18–64 years**	9.0 (8.3; 9.7)	8.3 (7.7; 8.9)	7.6 (7.1; 8.2)	8.0 (7.4; 8.6)	7.7 (7.2; 8.3)
Females aged 18–64 years	7.5 (6.7; 8.4)	6.7 (6.0; 7.5)	5.8 (5.2; 6.5)	6.2 (5.5; 6.9)	6.2 (5.6; 7.0)
Males aged 18–64 years	10.4 (9.4; 11.5)	9.8 (8.9; 10.8)	9.4 (8.5; 10.4)	9.8 (8.9; 10.7)	9.2 (8.4; 10.2)
**Both sexes by age group (years)**					
0–12	0.1 (0.0; 0.4)	0.1 (0.0; 0.5)	0.1 (0.0; 0.5)	0.1 (0.0; 0.4)	0
13–17	1.2 (0.6; 2.5)	1.2 (0.6; 2.4)	1.2 (0.6; 2.3)	1.5 (0.8; 2.7)	0.7 (0.3; 1.6)
18–19	5.4 (3.0; 9.8)	6.2 (3.7; 10.2)	5.5 (3.4; 8.9)	4.9 (2.9; 8.0)	3.5 (1.9; 6.3)
20–21	9.2 (5.9; 14.2)	10.2 (7.0; 15.1)	5.5 (3.4; 9.0)	8.8 (6.0; 12.9)	6.1 (3.9; 9.5)
22–23	9.3 (6.2; 14.0)	8.4 (5.6; 12.5)	9.6 (6.7; 13.9)	10.3 (7.3; 14.6)	11.3 (8.1; 15.6)
24–25	9.4 (6.3; 14.2)	11.2 (7.9; 15.9)	9.9 (7.0; 13.9)	7.2 (4.8; 10.7)	8.3 (5.7; 12.1)
26–29	11.6 (9.2; 14.7)	7.7 (5.8; 10.1)	9.2 (7.2; 11.7)	9.0 (7.1; 11.4)	8.3 (6.5; 10.6)
30–34	12.8 (10.5; 15.5)	10.9 (8.9; 13.3)	10.6 (8.7; 12.9)	10.0 (8.2; 12.3)	11.1 (9.2; 13.4)
35–39	8.6 (6.6; 11.0)	9.4 (7.5; 11.8)	10.4 (8.5; 12.8)	10.9 (8.9; 13.3)	9.0 (7.3; 11.2)
40–44	7.9 (6.2; 10.1)	9.0 (7.2; 11.2)	6.7 (5.2; 8.7)	7.7 (6.1; 9.9)	7.1 (5.6; 9.2)
45–49	8.4 (6.9; 10.3)	7.2 (5.9; 8.9)	6.7 (5.4; 8.3)	6.6 (5.3; 8.2)	6.6 (5.3; 8.4)
50–54	7.3 (5.8; 9.1)	6.2 (4.9; 7.8)	5.7 (4.5; 7.2)	6.7 (5.5; 8.3)	6.2 (5.0; 7.7)
55–59	7.6 (6.1; 9.6)	7.3 (5.8; 9.1)	5.2 (4.0; 6.7)	6.7 (5.3; 8.3)	6.1 (4.8; 7.6)
60–64	9.3 (7.4; 11.6)	7.7 (6.2; 9.8)	7.4 (5.9; 9.4)	7.2 (5.7; 9.1)	8.3 (6.7; 10.3)

Estimates of the incidence proportion of clozapine prescriptions are age- and sex-standardized to the population of Germany as of 31 December 2022.

Table S2 (continued): Standardized prescription incidence of clozapine by age and sex for each calendar year from 2012 to 2022 (per 100,000 persons; 95% confidence intervals in brackets)

	2017	2018	2019	2020	2021	2022
**Total number of (database) population, 0–64 years, n**	12,250,701	12,566,794	12,732,135	12,900,409	13,019,346	13,077,244
**Overall, 0–64 years**	5.6 (5.2; 6.1)	5.5 (5.1; 6.0)	5.4 (5.1; 5.9)	4.7 (4.3; 5.1)	4.7 (4.3; 5.1)	4.2 (3.8; 4.5)
Female, 0–64 years	4.6 (4.1; 5.2)	4.3 (3.8; 4.8)	4.4 (3.9; 4.9)	3.5 (3.1; 4.0)	3.6 (3.2; 4.1)	3.2 (2.8; 3.6)
Male, 0–64 years	6.6 (5.9; 7.3)	6.8 (6.1; 7.5)	6.5 (5.9; 7.2)	5.8 (5.3; 6.5)	5.8 (5.2; 6.4)	5.1 (4.6; 5.7)
**Both sexes aged 0–17 years**	0.3 (0.2; 0.7)	0.2 (0.1; 0.5)	0.3 (0.2; 0.6)	0.2 (0.1; 0.5)	0.3 (0.1; 0.6)	0.4 (0.2; 0.7)
Females aged 0–17 years	0.5 (0.2; 1.1)	0.2 (0.1; 0.7)	0.4 (0.2; 1.0)	0.4 (0.2; 1.0)	0.4 (0.2; 1.0)	0.2 (0.1; 0.8)
Males aged 0–17 years	0.2 (0.1; 0.7)	0.2 (0.1; 0.7)	0.2 (0.1; 0.7)	0.1 (0.0; 0.6)	0.2 (0.0; 0.6)	0.5 (0.2; 1.0)
**Both sexes aged 18–64 years**	7.1 (6.6; 7.6)	7.0 (6.5; 7.5)	6.9 (6.4; 7.4)	5.9 (5.5; 6.4)	5.9 (5.4; 6.4)	5.2 (4.8; 5.7)
Females aged 18–64 years	5.8 (5.1; 6.5)	5.3 (4.8; 6.0)	5.4 (4.8; 6.1)	4.3 (3.8; 4.9)	4.4 (3.9; 5.0)	4.0 (3.5; 4.6)
Males aged 18–64 years	8.3 (7.5; 9.2)	8.6 (7.8; 9.5)	8.2 (7.5; 9.1)	7.4 (6.7; 8.2)	7.3 (6.6; 8.1)	6.4 (5.7; 7.1)
**Both sexes by age group (years)**						
0–12	0	0	0	0.1 (0.0; 0.4)	0	0.1 (0.0; 0.4)
13–17	1.3 (0.7; 2.4)	0.8 (0.4; 1.9)	1.2 (0.6; 2.3)	0.7 (0.3; 1.7)	1.0 (0.5; 2.1)	1.2 (0.6; 2.4)
18–19	5.2 (3.2; 8.5)	4.2 (2.5; 7.3)	4.3 (2.5; 7.5)	4.9 (2.9; 8.2)	4.6 (2.7; 7.9)	4.3 (2.5; 7.6)
20–21	8.9 (6.2; 12.8)	7.1 (4.8; 10.6)	7.0 (4.6; 10.5)	7.1 (4.7; 10.7)	7.6 (5.1; 11.4)	4.6 (2.7; 7.8)
22–23	8.1 (5.5; 11.8)	7.0 (4.7; 10.4)	9.8 (7.0; 13.6)	7.2 (4.9; 10.6)	5.7 (3.7; 8.9)	7.9 (5.4; 11.5)
24–25	8.2 (5.6; 12.0)	8.9 (6.2; 12.7)	8.4 (5.8; 12.1)	5.9 (3.8; 9.0)	8.3 (5.8; 11.8)	8.1 (5.7; 11.6)
26–29	7.0 (5.4; 9.1)	8.9 (7.0; 11.2)	7.9 (6.1; 10.1)	8.4 (6.6; 10.7)	6.9 (5.3; 9.0)	5.5 (4.1; 7.4)
30–34	8.1 (6.5; 10.0)	8.4 (6.9; 10.4)	7.3 (5.9; 9.1)	7.0 (5.7; 8.7)	5.0 (3.9; 6.5)	5.0 (3.9; 6.5)
35–39	9.6 (7.9; 11.7)	7.2 (5.7; 9.0)	8.3 (6.7; 10.2)	5.8 (4.6; 7.4)	6.2 (4.9; 7.8)	5.6 (4.4; 7.2)
40–44	6.7 (5.2; 8.7)	6.7 (5.3; 8.7)	6.2 (4.8; 7.9)	5.8 (4.5; 7.5)	5.6 (4.4; 7.3)	4.9 (3.7; 6.4)
45–49	6.2 (4.9; 8.0)	6.9 (5.5; 8.7)	5.8 (4.5; 7.6)	5.0 (3.8; 6.7)	4.6 (3.4; 6.3)	3.4 (2.4; 4.8)
50–54	5.3 (4.2; 6.6)	6.2 (5.0; 7.7)	6.0 (4.8; 7.5)	4.7 (3.7; 6.1)	4.7 (3.6; 6.1)	4.9 (3.8; 6.3)
55–59	6.2 (5.0; 7.8)	6.3 (5.1; 7.8)	5.6 (4.5; 7.0)	4.3 (3.3; 5.5)	5.8 (4.6; 7.2)	4.6 (3.6; 5.9)
60–64	7.0 (5.5; 8.9)	6.1 (4.7; 7.7)	7.4 (5.9; 9.2)	6.7 (5.4; 8.4)	7.4 (6.0; 9.2)	6.1 (4.9; 7.7)

Estimates of the incidence proportion of clozapine prescriptions are age- and sex-standardized to the population of Germany as of 31 December 2022.

Table S3: Standardized prescription prevalence of clozapine (age 0–64 years) by regional characteristics for each calendar year from 2012 to 2022 (per 100,000 persons; 95% confidence intervals in brackets)

	**2012**	**2013**	**2014**	**2015**	**2016**
Total number of (database) population, n	11,650,141	12,130,060	12,372,493	12,658,252	12,733,756
**Overall**	77.6 (76.0; 79.3)	76.2 (74.6; 77.8)	74.9 (73.4; 76.5)	74.4 (72.8; 75.9)	74.5 (73.0; 76.0)
**Type of district according to settlement structure, i.e., urbanicity**					
Large urban city	94.7 (91.6; 98.0)	91.9 (88.9; 95.0)	90.3 (87.4; 93.4)	89.6 (86.7; 92.6)	90.2 (87.3; 93.2)
Urban district	74.6 (72.1; 77.3)	73.0 (70.6; 75.6)	71.7 (69.3; 74.2)	70.1 (67.8; 72.6)	69.9 (67.6; 72.3)
Rural district (with densification tendencies)	64.6 (60.8; 68.6)	65.4 (61.6; 69.4)	64.8 (61.1; 68.7)	66.5 (62.8; 70.4)	66.5 (62.7; 70.4)
Sparsely populated rural district	64.1 (60.1; 68.4)	64.8 (60.8; 69.1)	63.7 (59.7; 67.9)	63.4 (59.5; 67.6)	63.3 (59.5; 67.5)
**German Index of Socioeconomic Deprivation (2018)**					
1st quintile (least deprived)	88.7 (85.3; 92.3)	85.6 (82.3; 89.1)	81.9 (78.7; 85.3)	81.0 (77.8; 84.2)	81.4 (78.2; 84.6)
2nd to 4th quintile	74.7 (72.7; 76.8)	73.8 (71.8; 75.8)	73.4 (71.5; 75.4)	72.7 (70.8; 74.7)	73.0 (71.1; 75.0)
5th quintile (most deprived)	72.6 (68.5; 77.0)	72.0 (68.0; 76.3)	71.3 (67.3; 75.6)	71.5 (67.5; 75.7)	70.2 (66.3; 74.4)

Estimates of the prevalence proportion of clozapine prescriptions are age- and sex-standardized to the population of Germany as of 31 December 2022.

Table S3 (continued): Standardized prescription prevalence of clozapine (age 0–64 years) by regional characteristics for each calendar year from 2012 to 2022 (per 100,000 persons; 95% confidence intervals in brackets)

	**2017**	**2018**	**2019**	**2020**	**2021**	**2022**
Total number of (database) population, n	12,987,202	13,155,155	13,291,178	13,477,138	13,507,036	13,671,677
**Overall**	72.5 (71.1; 74.0)	72.0 (70.5; 73.5)	70.9 (69.4; 72.3)	68.6 (67.3; 70.1)	67.9 (66.5; 69.3)	65.5 (64.2; 66.9)
**Type of district according to settlement structure, i.e., urbanicity**						
Large urban city	85.9 (83.1; 88.8)	84.3 (81.6; 87.1)	81.5 (78.9; 84.3)	78.0 (75.4; 80.6)	76.3 (73.8; 78.9)	73.1 (70.7; 75.7)
Urban district	68.4 (66.1; 70.7)	68.1 (65.8; 70.4)	67.3 (65.1; 69.6)	65.5 (63.3; 67.7)	64.6 (62.4; 66.8)	63.0 (60.9; 65.2)
Rural district (with densification tendencies)	67.7 (64.0; 71.6)	67.3 (63.6; 71.2)	67.0 (63.3; 70.9)	65.5 (61.9; 69.3)	65.3 (61.7; 69.1)	62.4 (58.9; 66.1)
Sparsely populated rural district	62.0 (58.2; 66.1)	63.2 (59.3; 67.3)	64.3 (60.5; 68.5)	63.9 (60.1; 68.0)	65.6 (61.7; 69.8)	63.0 (59.2; 67.1)
**German Index of Socioeconomic Deprivation (2018)**						
1st quintile (least deprived)	78.9 (75.9; 82.1)	77.7 (74.8; 80.8)	75.6 (72.7; 78.6)	73.4 (70.6; 76.3)	71.4 (68.7; 74.3)	69.2 (66.5; 72.0)
2nd to 4th quintile	71.3 (69.5; 73.2)	70.8 (69.0; 72.7)	69.7 (67.9; 71.6)	67.4 (65.6; 69.2)	66.9 (65.2; 68.7)	64.2 (62.5; 65.9)
5th quintile (most deprived)	67.7 (63.9; 71.8)	68.1 (64.3; 72.2)	68.6 (64.8; 72.7)	66.8 (63.0; 70.9)	67.2 (63.4; 71.3)	66.2 (62.5; 70.2)

Estimates of the prevalence proportion of clozapine prescriptions are age- and sex-standardized to the population of Germany as of 31 December 2022.

Table S4: Prescription prevalence of clozapine (age 0–64 years) by district among the 202 districts with a database population of ≥20,000 persons in 2022 (per 100,000 persons), in ascending order of the standardized prevalence

District ID	Name of district	Urbanicity	Federal state	Database population, n	Clozapine users, n	Crude prevalence	Standardized prevalence	95% CI
3458	Oldenburg	Rural district (with densification tendencies)	Niedersachsen	32512	2	6.2	5.4	(1.4; 21.7)
9775	Neu-Ulm	Urban district	Bayern	26560	5	18.8	17.9	(7.4; 43.0)
3457	Leer	Rural district (with densification tendencies)	Niedersachsen	33446	7	20.9	19.9	(9.5; 41.7)
12065	Oberhavel	Sparsely populated rural district	Brandenburg	43065	10	23.2	21.5	(11.5; 40.2)
3461	Wesermarsch	Sparsely populated rural district	Niedersachsen	23382	6	25.7	22.6	(10.1; 51.0)
15003	Magdeburg, Stadt	Large urban city	Sachsen-Anhalt	30246	7	23.1	23.4	(11.0; 49.7)
7138	Neuwied	Urban district	Rheinland-Pfalz	35915	9	25.1	24.1	(12.5; 46.5)
9572	Erlangen-Höchstadt	Urban district	Bayern	24326	6	24.7	26.5	(11.9; 59.3)
9574	Nürnberger Land	Urban district	Bayern	25689	7	27.2	27.7	(13.1; 58.7)
12061	Dahme-Spreewald	Sparsely populated rural district	Brandenburg	33317	9	27.0	28.7	(14.5; 56.5)
9772	Augsburg	Urban district	Bayern	38279	11	28.7	29.7	(16.5; 53.6)
12069	Potsdam-Mittelmark	Rural district (with densification tendencies)	Brandenburg	42706	13	30.4	29.8	(17.1; 52.0)
5978	Unna	Urban district	Nordrhein-Westfalen	60526	19	31.4	29.9	(19.0; 47.1)
3451	Ammerland	Urban district	Niedersachsen	27139	8	29.5	31.1	(15.5; 62.5)
3453	Cloppenburg	Rural district (with densification tendencies)	Niedersachsen	41170	13	31.6	31.5	(18.3; 54.2)
7133	Bad Kreuznach	Rural district (with densification tendencies)	Rheinland-Pfalz	24822	9	36.3	31.6	(16.3; 61.4)
7143	Westerwaldkreis	Urban district	Rheinland-Pfalz	36772	11	29.9	31.6	(17.4; 57.4)
9175	Ebersberg	Urban district	Bayern	27644	9	32.6	31.7	(16.3; 61.3)
3361	Verden	Rural district (with densification tendencies)	Niedersachsen	33193	12	36.2	33.1	(18.7; 58.7)
12054	Potsdam, Stadt	Large urban city	Brandenburg	44103	14	31.7	33.2	(19.4; 56.7)
3459	Osnabrück	Rural district (with densification tendencies)	Niedersachsen	60392	19	31.5	33.5	(21.3; 52.5)
6633	Kassel	Urban district	Hessen	45727	15	32.8	34.6	(20.8; 57.5)
1062	Stormarn	Urban district	Schleswig-Holstein	76313	25	32.8	34.8	(23.4; 51.8)

District ID	Name of district	Urbanicity	Federal state	Database population, n	Clozapine users, n	Crude prevalence	Standardized prevalence	95% CI
5554	Borken	Urban district	Nordrhein-Westfalen	71615	26	36.3	35.5	(24.1; 52.2)
13072	Landkreis Rostock	Sparsely populated rural district	Mecklenburg-Vorpommern	48661	18	37.0	35.5	(22.2; 56.8)
5378	Rheinisch-Berg. Kr.	Urban district	Nordrhein-Westfalen	47799	17	35.6	35.9	(22.3; 57.9)
3356	Osterholz	Urban district	Niedersachsen	29821	10	33.5	36.0	(19.2; 67.7)
5170	Wesel	Urban district	Nordrhein-Westfalen	70815	26	36.7	36.5	(24.8; 53.8)
5913	Dortmund, Stadt	Large urban city	Nordrhein-Westfalen	103180	38	36.8	36.7	(26.6; 50.5)
9671	Aschaffenburg	Urban district	Bayern	29424	11	37.4	36.8	(20.3; 66.8)
3403	Oldenburg, Stadt	Large urban city	Niedersachsen	38902	15	38.6	36.9	(22.2; 61.3)
5954	Ennepe-Ruhr-Kreis	Urban district	Nordrhein-Westfalen	59096	23	38.9	37.0	(24.5; 55.8)
7132	Altenkir. (Westerw.)	Urban district	Rheinland-Pfalz	21020	8	38.1	37.2	(18.5; 74.8)
3455	Friesland	Urban district	Niedersachsen	21321	8	37.5	37.3	(18.4; 75.7)
6631	Fulda	Rural district (with densification tendencies)	Hessen	46700	17	36.4	37.3	(23.2; 60.2)
5374	Oberbergischer Kreis	Urban district	Nordrhein-Westfalen	42801	16	37.4	37.9	(23.2; 62.0)
13075	Vorp.-Greifswald	Sparsely populated rural district	Mecklenburg-Vorpommern	41079	16	38.9	38.5	(23.2; 63.8)
5117	Mülheim a.d.R., St.	Large urban city	Nordrhein-Westfalen	29307	11	37.5	38.6	(21.4; 69.9)
3353	Harburg	Urban district	Niedersachsen	66650	26	39.0	38.9	(26.4; 57.2)
9188	Starnberg	Urban district	Bayern	20495	7	34.2	38.9	(18.4; 82.1)
3352	Cuxhaven	Sparsely populated rural district	Niedersachsen	42360	18	42.5	39.0	(24.4; 62.2)
3359	Stade	Rural district (with densification tendencies)	Niedersachsen	45048	17	37.7	39.2	(24.4; 63.3)
6434	Hochtaunuskreis	Urban district	Hessen	55186	22	39.9	39.4	(25.8; 60.3)
1054	Nordfriesland	Sparsely populated rural district	Schleswig-Holstein	28840	11	38.1	39.8	(21.6; 73.5)
9178	Freising	Urban district	Bayern	29361	12	40.9	39.8	(22.6; 70.3)
5316	Leverkusen, Stadt	Large urban city	Nordrhein-Westfalen	20800	8	38.5	40.1	(19.9; 80.5)
3452	Aurich	Rural district (with densification tendencies)	Niedersachsen	26792	11	41.1	40.6	(22.5; 73.4)
13074	Nordwestmecklenburg	Rural district (with densification tendencies)	Mecklenburg-Vorpommern	29767	11	37.0	40.6	(22.2; 74.3)
6433	Groß-Gerau	Urban district	Hessen	48872	20	40.9	41.5	(26.7; 64.3)
9563	Fürth, Stadt	Large urban city	Bayern	20097	9	44.8	42.0	(21.8; 81.1)

District ID	Name of district	Urbanicity	Federal state	Database population, n	Clozapine users, n	Crude prevalence	Standardized prevalence	95% CI
13076	Ludwigslust-Parchim	Sparsely populated rural district	Mecklenburg-Vorpommern	36722	16	43.6	42.3	(25.6; 70.0)
9179	Fürstenfeldbruck	Urban district	Bayern	38996	16	41.0	43.1	(26.3; 70.6)
8426	Biberach	Rural district (with densification tendencies)	Baden-Württemberg	21710	10	46.1	43.3	(23.2; 80.9)
12064	Märkisch-Oderland	Sparsely populated rural district	Brandenburg	38538	17	44.1	43.6	(26.7; 71.1)
5362	Rhein-Erft-Kreis	Urban district	Nordrhein-Westfalen	87060	39	44.8	43.9	(32.0; 60.1)
12067	Oder-Spree	Sparsely populated rural district	Brandenburg	29208	14	47.9	43.9	(25.9; 74.3)
12063	Havelland	Sparsely populated rural district	Brandenburg	31988	13	40.6	44.0	(25.0; 77.4)
3151	Gifhorn	Sparsely populated rural district	Niedersachsen	20034	8	39.9	44.4	(21.9; 89.8)
13073	Vorpommern-Rügen	Sparsely populated rural district	Mecklenburg-Vorpommern	36825	17	46.2	44.5	(27.6; 71.8)
5119	Oberhausen, Stadt	Large urban city	Nordrhein-Westfalen	26335	12	45.6	44.6	(25.3; 78.5)
5758	Herford	Urban district	Nordrhein-Westfalen	28539	13	45.6	44.6	(25.8; 77.0)
3454	Emsland	Sparsely populated rural district	Niedersachsen	47688	22	46.1	44.8	(29.5; 68.0)
9174	Dachau	Urban district	Bayern	21368	9	42.1	45.4	(23.5; 87.9)
3251	Diepholz	Rural district (with densification tendencies)	Niedersachsen	43850	20	45.6	45.5	(29.2; 70.9)
6438	Offenbach	Urban district	Hessen	70570	33	46.8	45.8	(32.6; 64.6)
12072	Teltow-Fläming	Sparsely populated rural district	Brandenburg	31270	10	32.0	45.9	(23.8; 88.2)
1053	Herzogtum Lauenburg	Rural district (with densification tendencies)	Schleswig-Holstein	47288	22	46.5	46.3	(30.4; 70.7)
5112	Duisburg, Stadt	Large urban city	Nordrhein-Westfalen	66150	32	48.4	46.6	(32.9; 65.9)
6533	Limburg-Weilburg	Urban district	Hessen	38298	18	47.0	46.9	(29.4; 74.6)
5558	Coesfeld	Urban district	Nordrhein-Westfalen	48129	24	49.9	47.4	(31.6; 71.0)
5770	Minden-Lübbecke	Urban district	Nordrhein-Westfalen	37829	19	50.2	47.4	(30.0; 74.8)
5754	Gütersloh	Urban district	Nordrhein-Westfalen	42488	21	49.4	47.5	(30.8; 73.1)
5974	Soest	Urban district	Nordrhein-Westfalen	57064	28	49.1	47.6	(32.8; 69.1)
6435	Main-Kinzig-Kreis	Urban district	Hessen	81480	40	49.1	48.7	(35.7; 66.5)
5114	Krefeld, Stadt	Large urban city	Nordrhein-Westfalen	36760	18	49.0	49.3	(31.1; 78.4)
8118	Ludwigsburg	Urban district	Baden-Württemberg	77402	38	49.1	50.0	(36.3; 68.8)
5513	Gelsenkirchen, Stadt	Large urban city	Nordrhein-Westfalen	31326	16	51.1	50.3	(30.8; 82.1)

District ID	Name of district	Urbanicity	Federal state	Database population, n	Clozapine users, n	Crude prevalence	Standardized prevalence	95% CI
7141	Rhein-Lahn-Kreis	Urban district	Rheinland-Pfalz	22913	11	48.0	50.4	(27.7; 91.7)
5915	Hamm, Stadt	Large urban city	Nordrhein-Westfalen	21870	11	50.3	50.6	(27.9; 91.7)
1058	Rendsburg-Eckernförde	Rural district (with densification tendencies)	Schleswig-Holstein	68843	35	50.8	51.2	(36.6; 71.6)
12060	Barnim	Rural district (with densification tendencies)	Brandenburg	35217	18	51.1	51.3	(31.9; 82.6)
9184	München	Urban district	Bayern	71312	34	47.7	51.6	(36.7; 72.6)
1060	Segeberg	Rural district (with densification tendencies)	Schleswig-Holstein	70401	37	52.6	52.6	(38.1; 72.8)
8115	Böblingen	Urban district	Baden-Württemberg	50013	26	52.0	52.9	(36.0; 77.9)
5382	Rhein-Sieg-Kreis	Urban district	Nordrhein-Westfalen	127708	67	52.5	53.1	(41.8; 67.6)
5962	Märkischer Kreis	Urban district	Nordrhein-Westfalen	63278	35	55.3	53.5	(38.2; 74.9)
9576	Roth	Rural district (with densification tendencies)	Bayern	23716	13	54.8	53.5	(30.9; 92.6)
8136	Ostalbkreis	Urban district	Baden-Württemberg	26663	14	52.5	53.9	(31.8; 91.2)
8116	Esslingen	Urban district	Baden-Württemberg	62692	34	54.2	54.0	(38.5; 75.8)
5158	Mettmann	Urban district	Nordrhein-Westfalen	75696	42	55.5	54.6	(40.2; 74.1)
5370	Heinsberg	Urban district	Nordrhein-Westfalen	37317	21	56.3	54.8	(35.5; 84.4)
3241	Region Hannover	Urban district	Niedersachsen	190741	107	56.1	55.5	(45.9; 67.1)
5116	Mönchengladbach, St.	Large urban city	Nordrhein-Westfalen	41803	23	55.0	55.5	(36.9; 83.6)
7131	Ahrweiler	Rural district (with densification tendencies)	Rheinland-Pfalz	21874	12	54.9	55.8	(31.5; 98.7)
5166	Viersen	Urban district	Nordrhein-Westfalen	51599	29	56.2	56.1	(38.7; 81.2)
5570	Warendorf	Urban district	Nordrhein-Westfalen	45446	26	57.2	56.2	(38.2; 82.7)
3355	Lüneburg	Sparsely populated rural district	Niedersachsen	34645	20	57.7	56.4	(36.3; 87.7)
6436	Main-Taunus-Kreis	Urban district	Hessen	62538	36	57.6	56.5	(40.6; 78.5)
5154	Kleve	Urban district	Nordrhein-Westfalen	48381	27	55.8	57.1	(38.9; 83.7)
3254	Hildesheim	Urban district	Niedersachsen	43116	24	55.7	57.3	(38.3; 85.6)
7137	Mayen-Koblenz	Urban district	Rheinland-Pfalz	31243	18	57.6	57.4	(36.0; 91.5)
3351	Celle	Sparsely populated rural district	Niedersachsen	24610	14	56.9	58.3	(34.4; 98.8)
6432	Darmstadt-Dieburg	Urban district	Hessen	65539	39	59.5	58.3	(42.5; 80.0)
1056	Pinneberg	Urban district	Schleswig-Holstein	91892	55	59.9	58.4	(44.8; 76.2)

District ID	Name of district	Urbanicity	Federal state	Database population, n	Clozapine users, n	Crude prevalence	Standardized prevalence	95% CI
6440	Wetteraukreis	Urban district	Hessen	70582	42	59.5	58.6	(43.2; 79.4)
9187	Rosenheim	Urban district	Bayern	39713	22	55.4	58.6	(38.4; 89.5)
5162	Rhein-Kreis Neuss	Urban district	Nordrhein-Westfalen	81279	46	56.6	58.7	(43.9; 78.5)
1057	Plön	Rural district (with densification tendencies)	Schleswig-Holstein	31379	19	60.6	58.8	(37.2; 92.9)
8236	Enzkreis	Urban district	Baden-Württemberg	29729	17	57.2	58.9	(36.5; 94.9)
6611	Kassel, Stadt	Large urban city	Hessen	35621	20	56.1	59.0	(37.9; 91.7)
6412	Frankfurt a.M., Stadt	Large urban city	Hessen	170647	92	53.9	59.3	(48.0; 73.3)
9375	Regensburg	Rural district (with densification tendencies)	Bayern	23362	13	55.6	59.5	(34.4; 102.9)
6439	Rheingau-Taunus-Kreis	Urban district	Hessen	45394	27	59.5	60.3	(41.2; 88.4)
13003	Rostock, Stadt	Large urban city	Mecklenburg-Vorpommern	30592	20	65.4	60.8	(39.0; 94.7)
5315	Köln, Stadt	Large urban city	Nordrhein-Westfalen	251013	145	57.8	62.0	(52.4; 73.3)
2000	Hamburg, Stadt	Large urban city	Hamburg	562324	327	58.2	62.1	(55.6; 69.4)
5111	Düsseldorf, Stadt	Large urban city	Nordrhein-Westfalen	131220	77	58.7	62.1	(49.4; 78.1)
5911	Bochum, Stadt	Large urban city	Nordrhein-Westfalen	66198	41	61.9	64.3	(47.2; 87.5)
8117	Göppingen	Urban district	Baden-Württemberg	22517	15	66.6	64.6	(38.9; 107.3)
9761	Augsburg, Stadt	Large urban city	Bayern	42141	24	57.0	64.8	(43.0; 97.7)
7339	Mainz-Bingen	Urban district	Rheinland-Pfalz	46280	31	67.0	65.1	(45.6; 92.8)
13071	Mecklenburg. Seenpl.	Sparsely populated rural district	Mecklenburg-Vorpommern	39610	25	63.1	65.1	(43.3; 97.9)
6532	Lahn-Dill-Kreis	Urban district	Hessen	60131	39	64.9	65.4	(47.7; 89.6)
8111	Stuttgart, Stadt	Large urban city	Baden-Württemberg	97020	57	58.8	65.7	(50.2; 86.1)
5358	Düren	Urban district	Nordrhein-Westfalen	43559	28	64.3	65.8	(45.4; 95.4)
8119	Rems-Murr-Kreis	Urban district	Baden-Württemberg	48601	31	63.8	65.8	(46.2; 93.7)
9564	Nürnberg, Stadt	Large urban city	Bayern	77781	49	63.0	65.9	(49.7; 87.5)
7338	Rhein-Pfalz-Kreis	Urban district	Rheinland-Pfalz	27495	17	61.8	66.5	(41.2; 107.4)
5914	Hagen, Stadt	Large urban city	Nordrhein-Westfalen	30510	21	68.8	67.1	(43.7; 102.9)
3252	Hameln-Pyrmont	Rural district (with densification tendencies)	Niedersachsen	21568	14	64.9	68.8	(40.4; 117.4)
5562	Recklinghausen	Urban district	Nordrhein-Westfalen	86916	62	71.3	68.8	(53.6; 88.3)

District ID	Name of district	Urbanicity	Federal state	Database population, n	Clozapine users, n	Crude prevalence	Standardized prevalence	95% CI
8417	Zollernalbkreis	Urban district	Baden-Württemberg	20971	16	76.3	69.3	(42.1; 114.0)
3257	Schaumburg	Urban district	Niedersachsen	20165	14	69.4	69.6	(40.9; 118.5)
14713	Leipzig, Stadt	Large urban city	Sachsen	65404	47	71.9	69.7	(51.3; 94.7)
6411	Darmstadt, Stadt	Large urban city	Hessen	43115	27	62.6	69.8	(47.3; 103.0)
6414	Wiesbaden, Stadt	Large urban city	Hessen	62565	44	70.3	70.3	(52.2; 94.5)
11000	Berlin, Stadt	Large urban city	Berlin	1013019	680	67.1	70.3	(65.0; 75.9)
8125	Heilbronn	Urban district	Baden-Württemberg	34025	24	70.5	70.7	(47.4; 105.5)
4011	Bremen, Stadt	Large urban city	Bremen	253163	171	67.5	71.0	(61.1; 82.5)
8216	Rastatt	Urban district	Baden-Württemberg	33898	25	73.8	71.9	(48.4; 107.0)
8416	Tübingen	Urban district	Baden-Württemberg	36361	24	66.0	72.4	(48.2; 108.9)
9679	Würzburg	Urban district	Bayern	30718	21	68.4	73.4	(47.7; 113.1)
8336	Lörrach	Urban district	Baden-Württemberg	33827	25	73.9	73.7	(49.6; 109.5)
5366	Euskirchen	Urban district	Nordrhein-Westfalen	28970	22	75.9	74.3	(48.7; 113.5)
3101	Braunschweig, Stadt	Large urban city	Niedersachsen	49713	35	70.4	75.1	(53.6; 105.2)
6431	Bergstraße	Urban district	Hessen	51152	40	78.2	75.5	(55.3; 103.2)
5774	Paderborn	Urban district	Nordrhein-Westfalen	44468	33	74.2	76.1	(53.9; 107.3)
6634	Schwalm-Eder-Kreis	Rural district (with densification tendencies)	Hessen	28149	21	74.6	76.3	(49.5; 117.4)
7340	Südwestpfalz	Rural district (with densification tendencies)	Rheinland-Pfalz	25303	21	83.0	76.6	(49.4; 118.9)
8315	Breisg.-Hochschwarzw.	Urban district	Baden-Württemberg	41330	32	77.4	76.8	(54.1; 109.0)
10044	Saarlouis	Urban district	Saarland	24081	19	78.9	77.1	(49.0; 121.5)
16053	Jena, Stadt	Large urban city	Thüringen	21132	16	75.7	77.5	(46.5; 129.0)
14612	Dresden, Stadt	Large urban city	Sachsen	64530	48	74.4	78.2	(58.4; 104.6)
5113	Essen, Stadt	Large urban city	Nordrhein-Westfalen	95383	74	77.6	78.7	(62.5; 99.0)
7331	Alzey-Worms	Urban district	Rheinland-Pfalz	23536	20	85.0	79.7	(51.1; 124.3)
8215	Karlsruhe	Urban district	Baden-Württemberg	74891	62	82.8	80.3	(62.5; 103.2)
5766	Lippe	Urban district	Nordrhein-Westfalen	44936	37	82.3	82.1	(59.1; 114.0)
9162	München, Stadt	Large urban city	Bayern	324505	224	69.0	82.9	(72.2; 95.3)
6413	Offenbach a.M., Stadt	Large urban city	Hessen	22727	18	79.2	83.0	(51.9; 132.5)
1002	Kiel, Stadt	Large urban city	Schleswig-Holstein	52208	38	72.8	83.5	(60.2; 115.6)

District ID	Name of district	Urbanicity	Federal state	Database population, n	Clozapine users, n	Crude prevalence	Standardized prevalence	95% CI
5566	Steinfurt	Urban district	Nordrhein-Westfalen	83527	71	85.0	84.5	(66.9; 106.8)
7332	Bad Dürkheim	Urban district	Rheinland-Pfalz	25704	21	81.7	84.9	(55.1; 130.8)
5314	Bonn, Stadt	Large urban city	Nordrhein-Westfalen	81875	62	75.7	85.2	(66.1; 109.8)
5334	Städteregion Aachen	Urban district	Nordrhein-Westfalen	119637	93	77.7	85.9	(69.9; 105.5)
8316	Emmendingen	Urban district	Baden-Württemberg	23276	21	90.2	89.3	(58.0; 137.4)
3404	Osnabrück, Stadt	Large urban city	Niedersachsen	31621	28	88.5	90.0	(62.0; 130.9)
1059	Schleswig-Flensburg	Sparsely populated rural district	Schleswig-Holstein	42958	37	86.1	91.7	(66.3; 126.9)
16051	Erfurt, Stadt	Large urban city	Thüringen	22066	23	104.2	93.5	(62.1; 140.8)
1003	Lübeck, Stadt	Large urban city	Schleswig-Holstein	42693	40	93.7	94.0	(68.8; 128.5)
5124	Wuppertal, Stadt	Large urban city	Nordrhein-Westfalen	48435	43	88.8	94.3	(69.8; 127.2)
5711	Bielefeld, Stadt	Large urban city	Nordrhein-Westfalen	44174	40	90.6	94.3	(69.1; 128.7)
6534	Marburg-Biedenkopf	Rural district (with densification tendencies)	Hessen	53961	49	90.8	94.5	(71.3; 125.3)
6635	Waldeck-Frankenberg	Sparsely populated rural district	Hessen	27944	27	96.6	95.1	(64.7; 139.6)
5970	Siegen-Wittgenstein	Urban district	Nordrhein-Westfalen	40503	39	96.3	96.2	(70.1; 132.0)
8415	Reutlingen	Urban district	Baden-Württemberg	30199	31	102.7	97.1	(68.2; 138.2)
3357	Rotenburg (Wümme)	Sparsely populated rural district	Niedersachsen	28064	29	103.3	98.6	(68.2; 142.5)
6535	Vogelsbergkreis	Sparsely populated rural district	Hessen	20172	21	104.1	101.1	(65.8; 155.4)
1061	Steinburg	Sparsely populated rural district	Schleswig-Holstein	22436	24	107.0	104.0	(69.4; 155.7)
10041	Reg.verb. Saarbrücken	Urban district	Saarland	46283	49	105.9	105.0	(79.2; 139.3)
15002	Halle (Saale), Stadt	Large urban city	Sachsen-Anhalt	31376	34	108.4	107.0	(75.9; 150.7)
8317	Ortenaukreis	Urban district	Baden-Württemberg	61340	69	112.5	109.3	(86.1; 138.6)
3159	Göttingen	Urban district	Niedersachsen	58114	62	106.7	109.9	(85.6; 141.3)
5515	Münster, Stadt	Large urban city	Nordrhein-Westfalen	82690	84	101.6	114.7	(92.1; 142.7)
8212	Karlsruhe, Stadt	Large urban city	Baden-Württemberg	62287	67	107.6	116.9	(91.7; 149.1)
6531	Gießen	Urban district	Hessen	66878	78	116.6	117.5	(93.9; 147.1)
7315	Mainz, Stadt	Large urban city	Rheinland-Pfalz	53532	54	100.9	118.1	(89.9; 155.1)
8436	Ravensburg	Urban district	Baden-Württemberg	33547	41	122.2	118.5	(87.0; 161.4)
8311	Freiburg i.Br., Stadt	Large urban city	Baden-Württemberg	46690	49	104.9	120.3	(90.1; 160.7)
7337	Südliche Weinstraße	Urban district	Rheinland-Pfalz	24427	32	131.0	122.3	(85.9; 174.1)
8435	Bodenseekreis	Urban district	Baden-Württemberg	27976	34	121.5	125.6	(89.5; 176.3)

District ID	Name of district	Urbanicity	Federal state	Database population, n	Clozapine users, n	Crude prevalence	Standardized prevalence	95% CI
8222	Mannheim, Stadt	Large urban city	Baden-Württemberg	50274	59	117.4	126.7	(97.8; 164.3)
8226	Rhein-Neckar-Kreis	Urban district	Baden-Württemberg	103495	149	144.0	139.3	(118.5; 163.7)
4012	Bremerhaven, Stadt	Large urban city	Bremen	56893	76	133.6	139.6	(111.5; 174.8)
9190	Weilheim-Schongau	Rural district (with densification tendencies)	Bayern	21292	29	136.2	144.8	(100.6; 208.6)
1055	Ostholstein	Rural district (with densification tendencies)	Schleswig-Holstein	32055	46	143.5	147.5	(109.9; 198.0)
5958	Hochsauerlandkreis	Rural district (with densification tendencies)	Nordrhein-Westfalen	43327	68	156.9	152.2	(119.7; 193.4)
9173	Bad Tölz-Wolfratsh.	Sparsely populated rural district	Bayern	20595	31	150.5	154.5	(108.3; 220.5)
9362	Regensburg, Stadt	Large urban city	Bayern	24360	33	135.5	162.1	(113.8; 230.8)
8335	Konstanz	Urban district	Baden-Württemberg	51560	83	161.0	164.8	(132.7; 204.7)
8221	Heidelberg, Stadt	Large urban city	Baden-Württemberg	34844	46	132.0	166.8	(123.8; 224.7)
7314	Ludwigsh. a.R., Stadt	Large urban city	Rheinland-Pfalz	22578	44	194.9	208.4	(155.0; 280.2)
9663	Würzburg, Stadt	Large urban city	Bayern	25762	45	174.7	209.0	(154.5; 282.9)

CI, confidence interval.

Estimates of the prevalence proportion of clozapine prescriptions are age- and sex-standardized to the population of Germany as of 31 December 2022.

The sample included 202 (out of 401) districts with ≥20,000 individuals in the database population. Based on the total population of these districts in official statistics, they represent approximately 73% of the overall German population in 2022.
